# Metabolic engineering reveals *LUP5* as a determinant of saponin composition and insect feeding preference in *Barbarea vulgaris*

**DOI:** 10.1093/plphys/kiag291

**Published:** 2026-05-22

**Authors:** Jincheng Shen, Jan Günther, Sebastian Kjeldgaard-Nintemann, Pablo D Cárdenas, Søren Bak

**Affiliations:** Department of Plant and Environmental Sciences, Faculty of Science, University of Copenhagen, Frederiksberg C 1871, Denmark; Department of Plant and Environmental Sciences, Faculty of Science, University of Copenhagen, Frederiksberg C 1871, Denmark; Department of Plant and Environmental Sciences, Faculty of Science, University of Copenhagen, Frederiksberg C 1871, Denmark; Department of Plant and Environmental Sciences, Faculty of Science, University of Copenhagen, Frederiksberg C 1871, Denmark; Department of Plant and Environmental Sciences, Faculty of Science, University of Copenhagen, Frederiksberg C 1871, Denmark

## Abstract

Plant–insect coevolution has been a major driver of specialized metabolite diversification, yet the genetic basis of natural variation in defensive chemistry remains poorly understood. The wild crucifer winter cress (*Barbarea vulgaris*) comprises 2 ecotypes, an insect-resistant G-type and a susceptible P-type, characterized by distinct triterpenoid saponin profiles. To investigate the causal relationship between saponin composition and insect feeding preference, we established a stable transformation system for *B. vulgaris*. P-type *B. vulgaris* accumulates lupeol-derived saponins, and expression of the G-type β-amyrin synthase gene *LUP5* in the susceptible P-type conferred up to a 95% reduction in diamondback moth (*Plutella xylostella*) feeding, accompanied by increased accumulation of 3 hederagenin-derived monodesmosidic saponins. Comparison of *LUP5* expression driven by its native promoter and by the constitutive 35S promoter revealed that the native promoter is activated in young leaves, but not in young developing shoots, and leads to increased hederagenin accumulation in leaves. This expression pattern reflects the coordinated expression of downstream pathway genes and prevents expression in developing shoots. Our results provide direct in planta evidence that *LUP5* is a key determinant of natural variation in insect feeding preference in *B. vulgaris*, underscoring the pivotal role of the saponin backbone in herbivore deterrence. By linking promoter activity to metabolite structural diversity, this work provides mechanistic and conceptual insights into how plants coordinate specialized metabolism and defense.

## Teaser

A plant enzyme reshapes defensive chemicals and reduces insect feeding, revealing how small metabolic changes can alter herbivore behavior.

## Introduction

Plants have evolved a wide array of specialized metabolites to deter insect herbivores (Fürstenberg-Hägg et al. 2013; Yactayo-Chang et al. 2020; Beran and Petschenka 2022). Among these, triterpenoid saponins, which consist of a hydrophobic sapogenin backbone and one or more hydrophilic sugar moieties, represent a structurally diverse and ecologically important class of defensive compounds. Because of their structural similarity to membrane-embedded sterols, triterpenoid saponins interact with eukaryotic cell membranes (Augustin et al. 2011; Dervishi et al. 2026). In addition, their inherent bitterness may deter herbivores, contributing to their role as effective defense agents (Augustin et al. 2011). Thousands of triterpenoid saponins have been reported to date (Xu et al. 2004; Augustin et al. 2011; Moses et al. 2014; Huang et al. 2020). Due to their structural diversity, saponins have acquired a wide range of bioactivities, antimicrobial, and antifungal (Osbourn et al. 2011; Moses et al. 2014). Saponins can also be secreted from plant roots as exudates, where they mediate plant–microbe interactions and thereby contribute to plant development, further expanding their functional repertoire beyond defense (Tsuno et al. 2018; Fujimatsu et al. 2020). Importantly, specific saponin structures have been implicated in insect resistance, highlighting the functional significance of their chemical diversity (Stevenson et al. 2010; Cui et al. 2019; Hussain et al. 2019; Liu et al. 2019; Dolma et al. 2021).

The structure–activity relationships underlying saponin bioactivity are critical to their defensive function. For example, hederagenin 3-*O*-monoglucoside has both higher mortality and antifeedant activity than oleanolic acid 3-*O*-monoglucoside (which lacks C-23 hydroxylation), and gypsogenic acid 3-*O*-monoglucoside (which is further oxidized at C-23 to the ketone) (Liu et al. 2019). Additionally, monodesmosidic hederagenin and oleanolic acid exhibited higher mortality and antifeedant activity than their bidesmosidic counterparts (Tian et al. 2021; Dervishi et al. 2026). These observations suggest that even subtle modifications to the sapogenin backbone, glycosylation pattern, or sugar linkage can significantly alter saponin bioactivity. Despite growing knowledge of these structure–activity relationships, the genetic and biochemical mechanisms underlying saponin diversification remain poorly understood in most species.

One particularly promising ecological model to explore this question is the wild crucifer *Barbarea vulgaris*, which naturally segregates in 2 ecotypes or chemotypes that alter in both saponin profiles and insect resistance (Kuzina et al. 2011; Hauser et al. 2012; Byrne et al. 2017). The species comprises 2 ecotypes: the glabrous insect-resistant G-type, and the pubescent insect-susceptible P-type (Agerbirk et al. 2001). This intraspecific divergence likely arose from isolation in different Ice Age refugia, and now they naturally co-occur in Scandinavia (Hauser et al. 2012) and are characterized by marked differences in saponin composition (Kuzina et al. 2009; Khakimov et al. 2012, 2016). Gene duplication events have further contributed to the chemical divergence and insect feeding preference and mortality (Khakimov et al. 2015; Liu et al. 2019). Insect resistance in G-type *B. vulgaris* correlates with 4 oleanane-type defensive saponins, including oleanolic acid cellobioside, hederagenin cellobioside, gypsogenin cellobioside, and 4-epihederagenin cellobioside (Agerbirk et al. 2003; Kuzina et al. 2011). Among these, hederagenin cellobioside is particularly toxic and confers G-type insect resistance (Agerbirk et al. 2003; Nielsen et al. 2010; Kuzina et al. 2011).

**Figure 1:**
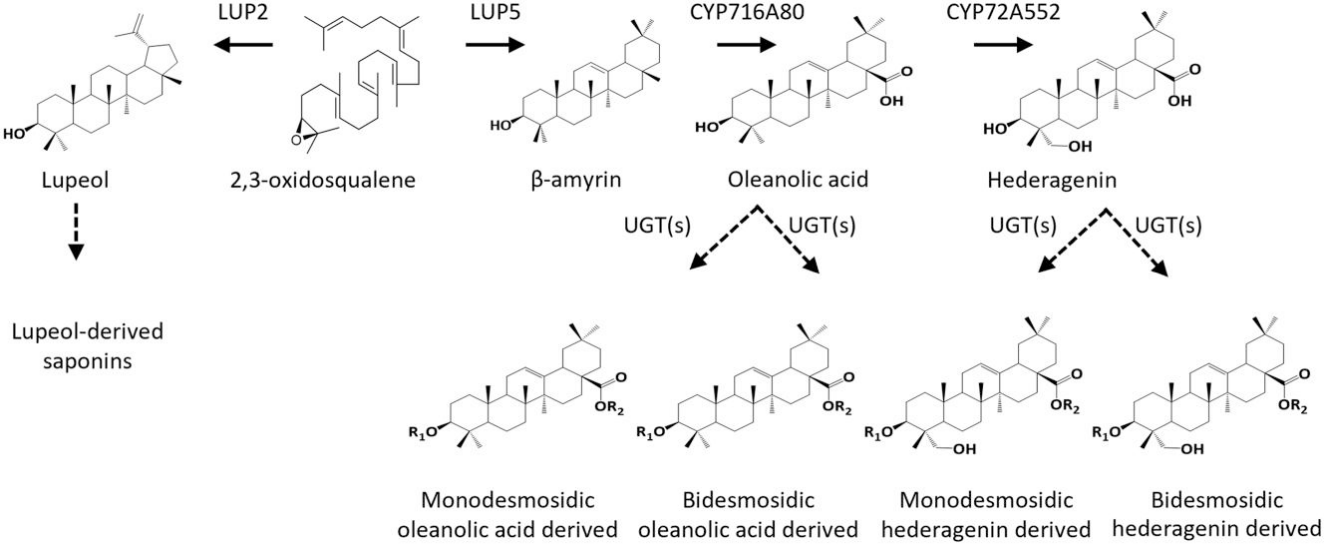
Lupeol and β-amyrin-derived triterpenoid saponin biosynthesis in *B. vulgaris* is derived from 2,3-oxidosqualene. In the P-type, *LUP2* prevails and lupeol-derived saponins accumulate, while in the G-type, *LUP5* prevails and β-amyrin-derived saponins accumulate. Variation in oxidosqualene cyclase product specificity therefore contributes to ecotype-specific differences in insect resistance. Monodesmosidic saponin, a single sugar chain is attached preferentially at C-3 (R1) or C-28 (R2), while for bidesmosidic saponin, sugar chains are attached at both C-3 (R1) and C-28 (R2). Dashed arrows indicate uncharacterized enzymatic steps, whereas solid arrows indicate functionally characterized steps.

The biosynthetic pathway leading to hederagenin cellobioside in G-type *B. vulgaris* has been elucidated up to the first glycosylation step (Fig. 1). The oxidosqualene cyclase *LUP5* is highly expressed in the G-type and primarily produces β-amyrin, while it in the P-type primarily produces α-amyrin (Khakimov et al. 2015; Günther et al. 2021). Subsequent enzymatic steps involve CYP716A80, CYP72A552, and UGT73C11, which sequentially convert β-amyrin into oleanolic acid, hederagenin, and their glycosylated derivatives (Fig. 1) (Augustin et al. 2012; Khakimov et al. 2015; Liu et al. 2019). The enzyme responsible for the final step in forming hederagenin cellobioside remains unidentified (Erthmann et al. 2018). In contrast, *LUP5* is expressed at very low levels in *B. vulgaris* P-type and primarily produces α-amyrin due to 2 amino acid substitutions as compared to the LUP5 in the G-type (Günther et al. 2021). Despite 98% sequence identity at the amino acid level, the G- and P-type LUP5 enzymes differ markedly in both their gene expression and product profiles. The P-type plants preferentially express *LUP2*, encoding for an oxidosqualene cyclase over 80% identical at the amino acid level to LUP5, but it produces predominantly lupeol (98%) and only 2% β-amyrin-derived saponins (Khakimov et al. 2015). Notably, the genes encoding for the remaining enzymes responsible for the biosynthesis of hederagenin 3-*O*-glucoside (CYP716A80, CYP72A552, and UGT73C11) are present in both P-type and G-type and exhibit similar substrate and product specificity (Augustin et al. 2012; Khakimov et al. 2015; Liu et al. 2019). However, the *LUP2* and *LUP5* genes in the 2 eco-types are differentially regulated, suggesting that upstream regulation and oxidosqualene cyclase product specificity may be key drivers of saponin structural and insect resistance differences.

To investigate the genetic and biochemical basis of saponin-mediated insect feeding preference, we leveraged the natural saponin variation in *B. vulgaris* to develop a model system that links in planta metabolic engineering of saponin composition with ecological function. We provide functional evidence that engineering saponin biosynthesis through manipulation of G-type *LUP5* expression substantially reduces insect feeding preference in this wild crucifer and explains the molecular basis of the difference in insect resistance between the G- and P-type *B. vulgaris*. By comparing constructs driven by the native *LUP5* and constitutive 35S promoters, we further reveal how promoter activity shapes metabolite structural diversity. These findings offer mechanistic insight into plant–insect coevolution and established a model system for dissecting genotype–phenotype relationships in plant chemical specialization.

## Results

### Establishment of a transformation and regeneration system in the wild crucifer *B. vulgaris*

To enable functional studies of insect preference feeding in the wild crucifer *B. vulgaris* as an ecological model system, we successfully established a stable transformation and regeneration method for both the G-type and P-type ecotypes (Fig.1, Figure S1, Table S10). To metabolically engineer the saponin compositions, the G-type *LUP5* was introduced into both ecotypes. In addition, we attempted to silence *CYP72A552* in the G-type using RNAi. G-type *LUP5* expression was driven either by the constitutive 35S promoter or by its 1,988 bp native *LUP5* promoter. Transformation efficiency, calculated as the percentage of positive transgenic plants to all obtained plants from tissue culture, reached up to 94% across experiments (Table 1). The native G-type *LUP5* promoter exhibited approximately 2-fold higher transformation efficiency than the 35S promoter in both the G- and P-types, indicating a selection pressure against *LUP5* overexpression from the 35S promoter (Table 1).

**Table 1:** Stable transformation system in *B. vulgaris* enabled up to 94% efficiency.

Construct for transformation	Ecotype	Total numbers of plants	Positive/ total (%)
pJCV51-pLUP5::LUP5	G-type	31	94
	P-type	35	60
pJCV51-p35S::LUP5	G-type	52	40
	P-type	66	26
pJCV51-pLUP5::eGFP	G-type	24	58
pJCV51-p35S::eGFP	G-type	20	55
pK7FWG2-CYP72A552 RNAi	G-type	46	28

### Stable expression of G-type *LUP5* in the P-type reduces insect feeding preference

**Figure 2:**
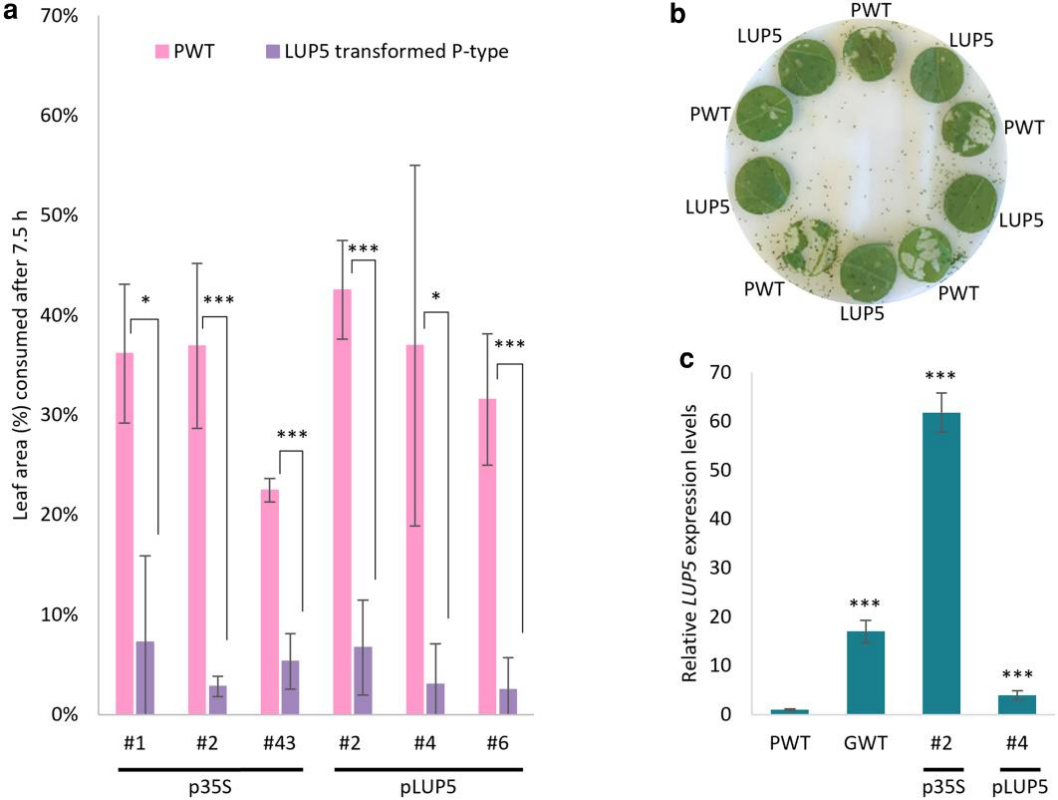
Feeding preference was significantly reduced in *LUP5*-transformed P-type *B. vulgaris* to *P. xylostella* larvae. a) Leaf area (%) consumed after 7.5 h (choice test, 3 individual plants per transgenic line; each replicate represents one Petri dish containing 5 transgenic and 5 wild-type leaf discs). PWT, wild-type P-type *B. vulgaris*; p35S, p35S::LUP5 transformed P-type *B. vulgaris*; pLUP5, pLUP5::LUP5 transformed P-type *B. vulgaris*. b) Example of the insect choice feeding assay, and the resulting leaf condition after 7.5 h insect exposure. LUP5, P-type *B. vulgaris* expressing the G-type *LUP5*. c) Relative *LUP5* expression levels, as determined by RT-qPCR (3 individual plants per transgenic line). Expression levels were normalized to *Tubulin* and are shown relative to the wild-type P-type control, which was set to 1. Numbers below the bars indicate independent transgenic lines. GWT, wild-type G-type *B. vulgaris*. Error bars represent the standard deviation of the mean from 3 individual plants (a and c). Statistical significance was determined using a paired *t*-test (a) and ANOVA (c) with wild-type P-type as the control. Asterisks indicate significant differences (*P < 0.05 and ***P < 0.005) (a and c).

To assess the effect of G-type *LUP5* expression on insect feeding preference across the transgenic lines (Table 1), we conducted a choice feeding assay in which third-instar *Plutella xylostella* larvae were presented with alternating wild-type and transgenic *B. vulgaris* leaf discs. Under natural conditions, the G- and P-types co-occur (Hauser et al. 2012), and accordingly, we tested in choice tests to better mimic the natural preference feeding selection. Leaf area consumed was quantified when approximately half of the total leaf material of the wild type had been eaten (approx. 7.5 h). The best performing *LUP5*-transformed P-type plants exhibited a significant reduction in larval consumption, ranging from 76% to 95% (Fig. 2, a and b). RT-qPCR on the best performing 35S and *LUP5* promoter lines confirmed that the G-type *LUP5* was transformed and expressed in the P-type both under the 35S promoter or the native G-type *LUP5* promoter (Fig. 2c). Interestingly, the 35S promoter expressed LUP*5* at approximately 16-fold higher levels than those in the best performing pLUP5::LUP5 transformed P-type and the wild-type P- and G-types (Fig. 2c).

### Expression of G-type *LUP5* induced β-amyrin-derived saponins in P-type *B. vulgaris*

Based on the highest reduction of leaf area consumed, the 3 best performing transgenic lines were selected for each promoter type (35S and native G-type *LUP5*) and subjected to untargeted and targeted LCMS analyses. The untargeted metabolite analysis revealed approximately 9,500 distinct *m*/*z* features, each defined as a unique *m*/*z* and retention time (RT) pair (Table S1). Features differing between *LUP5*-expressing and wild-type plants (*P* < 0.05) were ranked by fold change, and the top 100 increased and 100 decreased features were analyzed further. However, extraction of the *m*/*z* features using DataAnalysis software revealed that the majority lacked clear MS/MS fragmentation patterns, preventing structural annotation. Many of these features likely represent in-source fragments, background ions, or uncharacterized metabolites with inconclusive fragmentation (Figure S2).

**Figure 3:**
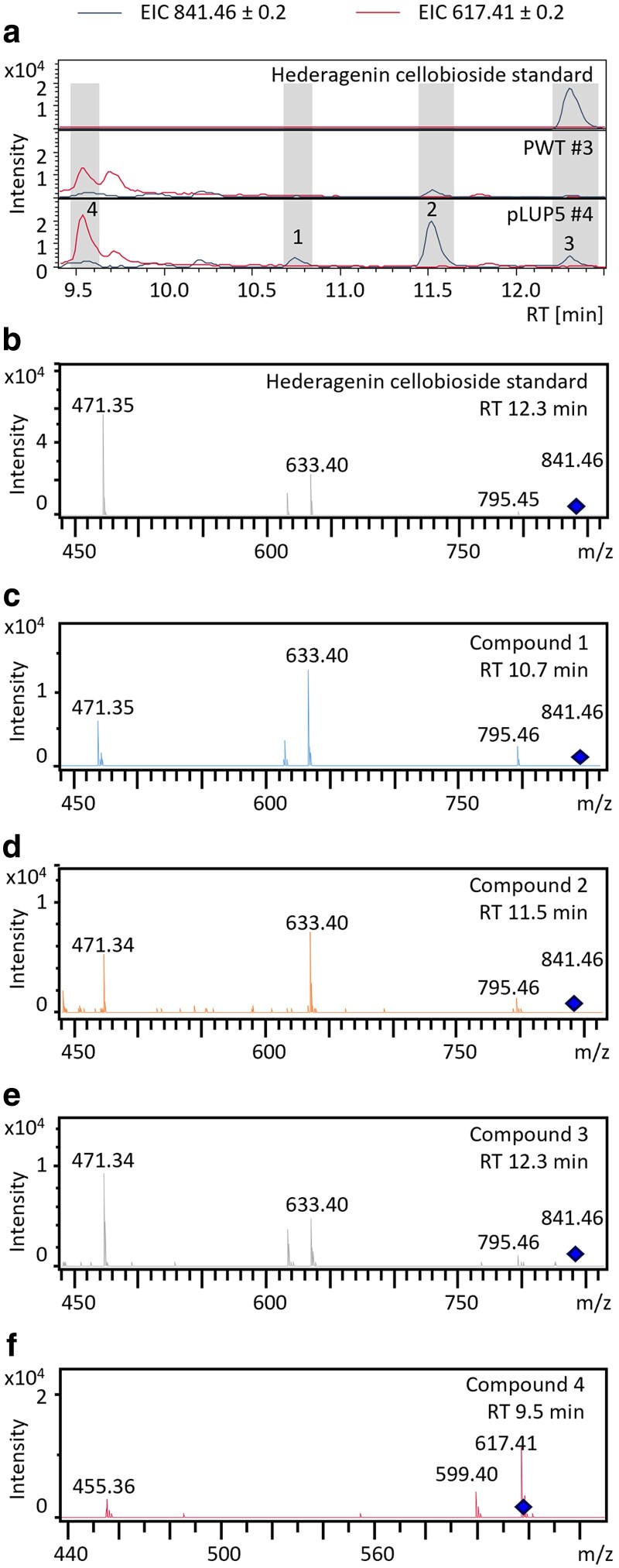
Glycosylated oleanolic acid and hederagenin levels were increased in P-type *B. vulgaris* transformed with G-type *LUP5*. Representative saponins were tentatively identified based on fragmentation patterns and retention times by comparison with standards and previously reported saponins, as shown in this figure. a) Extracted ion chromatograms of monodesmosidic hederagenin with 2 hexose units (*m/z* 841.46 ± 0.2) and oleanolic acid monoglucoside (*m/z* 617.41 ± 0.2). EIC, extracted ion chromatogram; PWT #3, wild-type P-type *B. vulgaris* line 3; pLUP5 #4, pLUP5::LUP5 transformed P-type *B. vulgaris* line 4; RT, retention time. b) Fragmentation pattern of the hederagenin cellobioside standard, retention time 12.3 min. c to e) Fragmentation patterns of monodesmosidic hederagenin with 2 hexose units, retention times at 10.7, 11.5, and 12.3 min in G-type *LUP5*-expressing P-type *B. vulgaris*. Compound 3 was identified as hederagenin cellobioside based on an identical fragmentation pattern and retention time to a hederagenin cellobioside standard analyzed in the same batch. f) Fragmentation pattern of oleanolic acid monoglucoside, retention time 9.5 min. Compound 4 showed the same fragmentation pattern as oleanolic acid 3-glucoside previously reported by Augustin et al. (2012).

Consequently, a targeted saponin analysis was conducted using the Bruker DataAnalysis software. *B. vulgaris* saponins were detected by extracted ion chromatograms of our previously tentatively identified aglycones, and their structures were inferred from characteristic fragmentation patterns. Based on 455, 469, and 471 *m*/*z*, the characteristic fragmentation patterns of oleanolic acid, gypsogenin, and hederagenin were tentatively identified, respectively (Fig. 3). This target approach identified 10 putative distinct saponins in P-type lines and 30 in G-type lines (Table S2 and S3). All detected saponins were classified as monodesmosidic, based on their fragmentation patterns. Saponin abundance was subsequently quantified by calculating peak areas from extracted ion chromatograms (Table S2 and S3).

**Figure 4:**
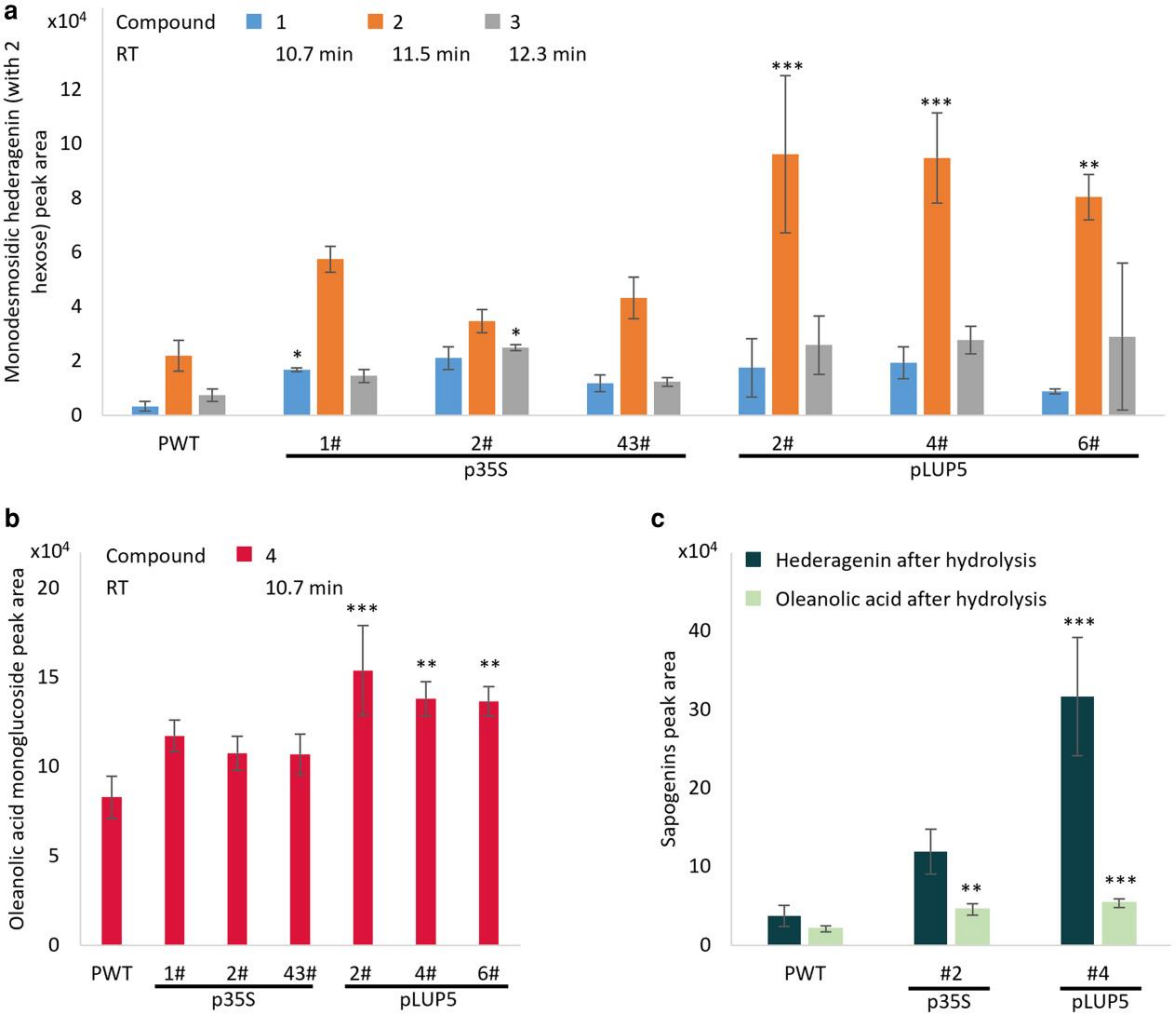
Native G-type *LUP5* promoter leads to higher levels of glycosylated oleanolic acid and hederagenin in *LUP5*-transformed P-type *B. vulgaris* than the constitutive 35S promoter. To evaluate the effect of G-type *LUP5* expression on saponins and their corresponding sapogenin backbone in P-type *B. vulgaris*, we tentatively identified and quantified saponin composition in leaf extracts using LC–MS/MS. a) Monodesmosidic hederagenin (with 2 hexoses) peak area (EIC: *m/z* = 841.46 ± 0.2). PWT, wild-type P-type *B. vulgaris*; p35S, p35S::LUP5 transformed P-type *B. vulgaris*; pLUP5, pLUP5::LUP5 transformed P-type *B. vulgaris*; RT, retention time. b) Oleanolic acid monoglucoside peak area (EIC: *m/z* = 617.41 ± 0.2). c) Peak area of sapogenins, including total hederagenin (EIC: *m/z* = 437.34 ± 0.2) and total oleanolic acid (EIC: *m/z* = 439.36 ± 0.2). Numbers below the bars indicate independent transgenic lines. Error bars represent the standard deviation of the mean from 3 individual plants. Statistical significance was determined using ANOVA with wild-type P-type as the control. Asterisks indicate significant differences (*P < 0.05, **P < 0.01, and ***P < 0.005).

The LCMS analysis revealed 3 hederagenin-derived monodesmosidic saponins (2 hexoses; RT 10.7, 11.5, and 12.3 min) and 1 oleanolic acid monoglucoside (1 hexose; RT 9.5 min) that accumulated at higher levels in both 35S- and native G-type *LUP5* promoter-driven *LUP5*-transformed P-type plants, compared to wild-type P-type (Fig. 3, a and b, Table 2). The most abundant of the 3 hederagenin-derived saponins eluted at 11.5 min and was increased ∼4-fold in the P-type expressing *LUP5* under the native G-type *LUP5* promoter compared to the wild-type and was ∼2-fold more abundant than in P-type plants transformed with *LUP5* under the 35S promoter (Fig. 4a). Hederagenin cellobioside (eluted at 12.3 min) was also increased ∼4-fold but remained at lower levels in the P-type expressing G-type *LUP5* under the native G-type *LUP5* promoter (Fig. 4a). These 3 hederagenin-derived saponins were also present in the control G-type plants, with hederagenin cellobioside (eluted at 12.3 min) being the most abundant hederagenin-derived saponin (Table S3 and S6). An increase of the oleanolic acid monoglucoside (eluted at 9.5 min) was not detected in G-type (Table S3). The other detected saponins remained unchanged (Table S4).

**Table 2:** Seven triterpenoid saponins were increased by G-type LUP5 expression either in G- or P-type.

Compound number	*Barbarea vulgaris*	Sapogenin	Sugar moieties	Formic acid	Fragmentation pattern in mass spectra	Retention time (min)
1	P- and G-types	Hederagenin	2 x hexose	Yes	[M841 − 46 − 162 − 162 = 471]	10.7
2	P- and G-types	Hederagenin	2 x hexose	Yes	[M841 − 46 − 162 − 162 = 471]	11.5
3	P- and G-types	Hederagenin	2 x hexose	Yes	[M841 − 46 − 162 − 162 = 471]	12.3
4	P-type	Oleanolic acid	1 x hexose	No	[M617 − 162 = 455]	9.5
5	G-type	Hederagenin	3 x hexose	Yes	[M1003 − 46 − 162 − 162 − 162 = 471]	8.5
6	G-type	Oleanolic acid	2 x hexose	No	[M779 − 162 − 162 = 455]	11.0
7	G-type	Oleanolic acid	3 x hexose	Yes	[M987 − 46 − 162 − 162 − 162 = 455]	10.7

Saponins were tentatively identified based on fragmentation patterns and retention times by comparison with standards and with previously reported saponins (Augustin et al. 2012; Khakimov et al. 2016). Compounds were detected in both P- and G-type *B. vulgaris* or were predominantly present in one ecotype.

To evaluate the effect of G-type *LUP5* expression on P-type *B. vulgaris* saponin aglycone biosynthesis and accumulation, levels of free and total sapogenins were analyzed. Free sapogenins were measured from untreated samples, representing aglycones occurring in a nonglycosylated form. Total sapogenins were defined as the sum of free and sugar-bound sapogenins, the latter released through hydrochloric acid-mediated hydrolysis of glycosylated saponins. A single transgenic line (with 3 vegetative clones) from both p35S::*LUP5* and pLUP5::*LUP5*-transformed P-type *B. vulgaris* was selected based on reduced leaf area consumption and increased accumulation of 4 saponins (Fig. 2a, Fig. 4, a and b, Table 2). This analysis revealed a significant increase in total hederagenin and oleanolic acid levels in *LUP5*-transformed P-type plants (Fig. 4c), while total lupeol, β-amyrin, and free sapogenins remained undetectable (Table S5). Higher accumulation of total hederagenin was observed in plants expressing *LUP5* under the native G-type *LUP5* promoter as compared to those under the 35S promoter (Fig. 4c).

**Figure 5:**
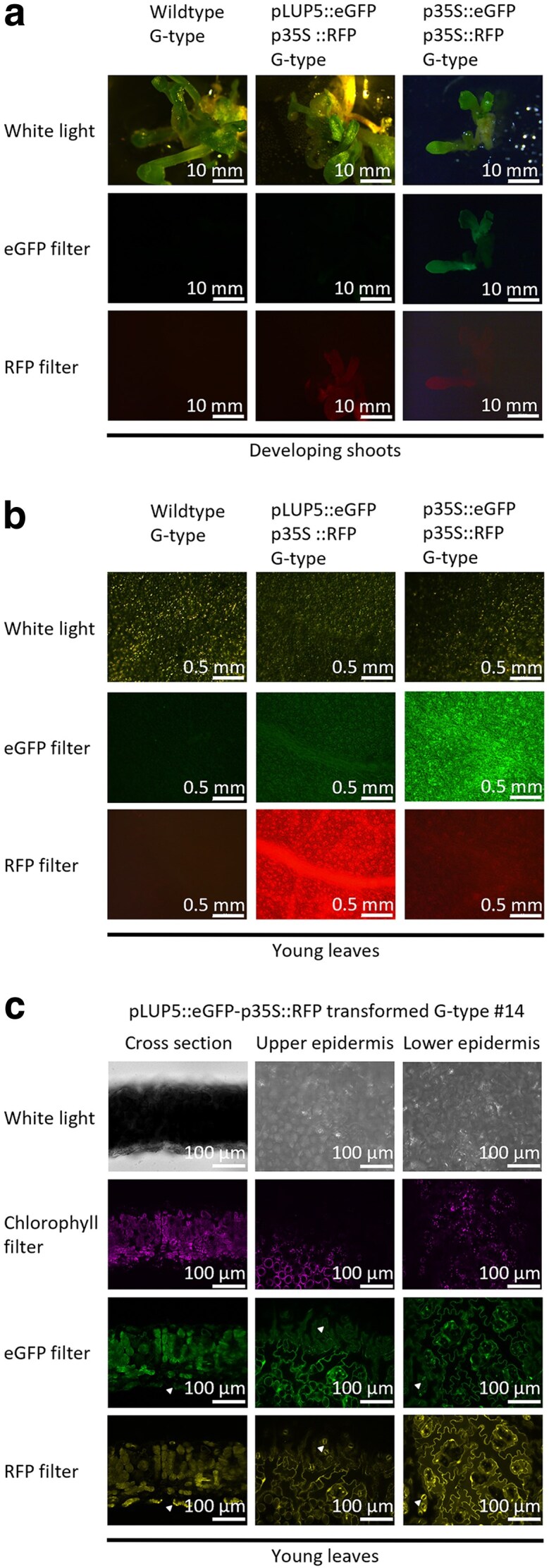
Native G-type *LUP5* promoter is active in young leaves but not in developing shoots. To examine differences between the native G-type *LUP5* and 35S constitutive promoters, we analyzed promoter activity and tissue-specific patterns of both promoters during plant regeneration and early growth. pLUP5::eGFP–p35S::RFP and p35S::eGFP–p35S::RFP constructs were stably transformed into G-type plants, and fluorescence signals were examined by stereomicroscopy (a, b) and confocal laser scanning microscopy (CLSM) (c). RFP, driven by the 35S promoter, was used as a selection marker and to compare promoter activity across tissues. a) During the shoot induction stage, the native G-type *LUP5* promoter showed weak or no activity, whereas the constitutive 35S promoter exhibited clear fluorescence. No detectable eGFP signal was observed in developing shoots on induction media carrying the pLUP5::eGFP–p35S::RFP construct. Scale bars, 10 mm. b) Young leaves examined at 4 weeks after root induction showed eGFP fluorescence under both the native G-type *LUP5* and constitutive 35S promoters, though the signal driven by the 35S promoter remained stronger. Scale bars, 0.5 mm. c) No clear leaf tissue-specific promoter activity was observed between the 35S and *LUP5* promoters. Leaf discs (upper and lower epidermis) or hand sections were imaged on a Leica SP8-X confocal laser scanning microscope equipped with a Fluotar VISIR 25x/0.95 WATER objective (Leica Microsystems). eGFP was excited at 488 nm and detected between 498 and 550 nm. RFP was excited at 584 nm and detected between 594 and 650 nm. Chlorophyl was detected between 650 and 720 nm. Weak or no GFP signal was detected in stomatal guard cells; the visible signal at the stomatal opening represents autofluorescence from the cuticle, as confirmed by comparison with the RFP channel. The pLUP5::eGFP–p35S::RFP line #14 is shown as a representative example. White triangles indicate stomata. Scale bars, 100 µm.

To investigate how promoter choice affects gene expression and saponin composition, we compared the previously described transgenic P-type lines expressing G-type *LUP5* under either the constitutive 35S or the native G-type *LUP5* promoter. Interestingly, although the 35S promoter drove much higher *LUP5* expression levels as compared to the native G-type *LUP5* promoter (Fig. 2c), this did not result in proportionally increased accumulation of β-amyrin-derived saponins (Fig. 4a to c). To investigate this, we generated eGFP lines with the native G-type *LUP5* and 35S promoter, respectively, to reveal the expression profile of the G-type *LUP5* promoter. This showed that while the 35S promoter, as expected, was active at the shoot induction stage and consistently drove strong fluorescence signals in young leaves, the native G-type *LUP5* promoter was not active in the shoot induction stage but induced expression in young leaves (Fig. 5, a to c). No distinct differences in tissue-specific promoter activity, with stomatal guard cells as the only exception, were observed between the 35S and native G-type *LUP5* promoters in young leaves from plants 4 weeks after root induction and soil transfer (Fig. 5c). These observations indicate that the 2 promoters differ mainly in temporal activation and relative expression strength, which likely contributes to the observed variation in transcript levels, metabolite accumulation, and transformation efficiency.

**Figure 6:**
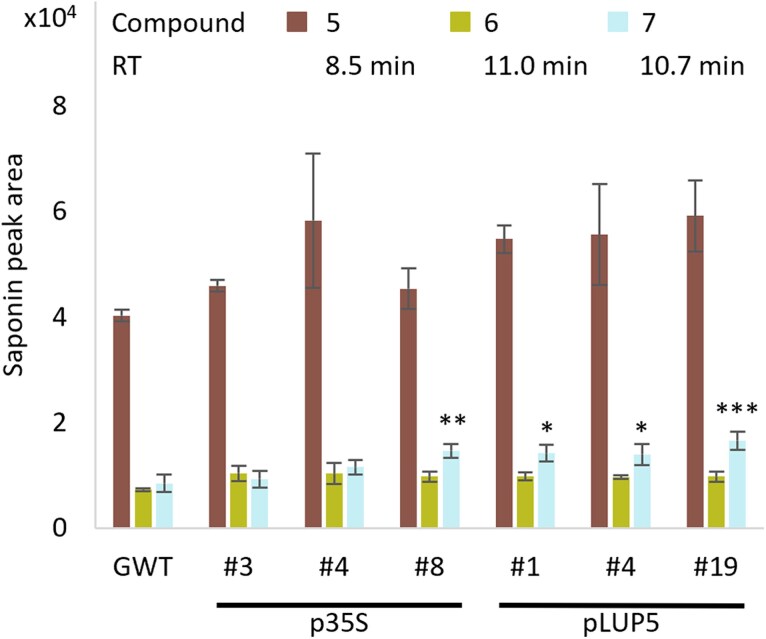
Glycosylated oleanolic acid and hederagenin levels are increased in G-type *B. vulgaris* expressing G-type *LUP5*. Based on the greatest increase in saponin level, the 3 best-performing transgenic lines were selected for each promoter type (35S and native G-type *LUP5*). Compounds 5 to 7 correspond to monodesmosidic hederagenin with 3 hexose units (EIC, *m/z* 1,003.51 ± 0.2), monodesmosidic oleanolic acid with 2 hexose units (EIC, *m/z* 617.41 ± 0.2), and monodesmosidic oleanolic acid with 3 hexose units (EIC, *m/z* 987.52 ± 0.2), respectively. Numbers below the bars indicate independent transgenic lines. GWT, wild-type G-type *B. vulgaris*; p35S, p35S::LUP5 transformed G-type *B. vulgaris*; pLUP5, pLUP5::LUP5 transformed G-type *B. vulgaris*. Error bars represent the standard deviation of the mean from 3 individual plants. Statistical significance was assessed using ANOVA with wild-type G-type as the control. Asterisks indicate significant differences (*P < 0.05, **P < 0.01, and ***P < 0.005).

Introduction of G-type *LUP5* in G-type increased a monodesmosidic hederagenin (with 3 hexoses) and an oleanolic acid monoglucoside (with 2 hexoses) in both p35S::LUP5 and pLUP5::LUP5 transformed G-type plants, RTs 8.5 and 11.0 min, respectively (Fig. 6, Table 2). Monodesmosidic oleanolic acid (with 3 hexose, RT at 10.7 min) increased in all pLUP5::LUP5 transformed G-type lines (Fig. 6). Twenty-six additional saponins remained unchanged (Table S6), and the 3 slightly elevated saponins remained at low abundance relative to other saponins (Fig. 6, Table S6).

In conclusion, stable transformation of G-type *LUP5* in P-type significantly increased 1 oleanolic acid monoglucoside (with 1 hexose) and 3 hederagenin-derived monodesmosidic saponins (each with 2 hexoses) by approximately 50% to 4-fold, depending on the promoter and saponin (Fig. 4, a and b). Notably, the oleanolic acid monoglucoside, eluting at 9.5 min, was predominantly present in the P-type (Fig. 4b, Table S3). The most abundant hederagenin-derived saponin in P-type eluted at 11.5 min, whereas hederagenin cellobioside (eluted at 12.3 min) remained the dominant saponin in G-type (Fig. 4a, Table S4 and S6). Introduction of G-type *LUP5* in G-type slightly increased saponin levels, but their concentrations remained low compared to other oleanolic acid- and hederagenin-derived compounds (Fig. 6, Table S6). Expression of G-type *LUP5* by its native G-type *LUP5* promoter resulted in higher transformation efficiency and induced greater accumulation of β-amyrin-derived sapogenins and their glycosylated saponins, despite lower LUP5 transcript levels compared to the 35S promoter (Fig. 4, a to c, Fig. 6, Table 1, Table S5).

### Reduced accumulation of hederagenin-derived saponins following *CYP72A552* silencing in G-type *B. vulgaris* does not alter insect feeding preference

To downregulate *CYP72A552* in G-type *B. vulgaris*, an RNAi construct targeting a 190 bp region of the gene was generated (Figure S4). Silencing of *CYP72A552* reduced the accumulation of 6 saponins in the G-type, including hederagenin monoglucoside and 3 additional hederagenin-derived monodesmosidic saponins (Figure S3, a to e, Supplementary Table S7). To evaluate the impact of G-type *CYP72A552* silencing on insect feeding preference, the 3 best performing lines showing the greatest reduction in hederagenin cellobioside levels were subjected to a preference feeding assay as described above. However, neither the transgenic G-type nor the wild-type G-type plants were consumed by *P. xylostella* larvae as measured after 7.5 h (Figure S3g). Thus, although *CYP72A552* silencing significantly reduced hederagenin biosynthesis up to 40% (Figure S3f) and the accumulation of its glycosylated derivatives, however, this did not lead to an induction in consumption by *P. xylostella* larvae.

## Discussion

Deciphering the genetic and biochemical bases of natural insect resistance is essential for our basic understanding of chemical ecology and for addressing global challenges in crop protection and sustainability. Our study contributes to this effort by elucidating how structural diversification in saponins mediates ecological defense.

Triterpenoid saponins are present in more than 100 plant families and are known as mediators of plant–insect interactions. They have attracted attention due to their high chemical diversity and structure-dependent bioactivities and unique modes of action. Owing to their structural similarity to sterols and steroid hormones, triterpenoid saponins are proposed to interact with eukaryotic cell membranes, which may underlie their diverse biological effects (Augustin et al. 2011). Key factors influencing insect resistance include the overall structure of the saponin, the composition of the sapogenin backbone, the types of sugar moieties, the length and number of glycoside chains, and the sites of glycosylation (Gao et al. 2011; Liu et al. 2019; Tian et al. 2021; Dervishi et al. 2026). Prior metabolic engineering efforts have focused on modifying saponin composition by disrupting biosynthetic genes in legumes (Confalonieri et al. 2021; Hodgins et al. 2024). For example, Confalonieri et al. (2021) knocked out *CYP93E2*, which hydroxylates β-amyrin at the C-24 position, in *Medicago truncatula*, resulting in transgenic lines that no longer produced nonhemolytic soyasapogenol saponins but instead redirected metabolic flux toward the synthesis of hemolytic saponins. Hodgins et al. (2024) knocked out β-amyrin synthase *BAS1* in pea (*Pisum sativum*), leading to a 99.8% reduction in saponin content in the seeds. However, these studies did not address how targeted saponin engineering influences ecological traits such as herbivore preference or consumption.

We, and others, have previously transiently expressed saponin biosynthetic genes in *Nicotiana benthamiana* (Khakimov et al. 2015; Liu et al. 2019; Reed et al. 2023). An unexpected drawback of the transient tobacco system was that hederagenin was further metabolized by endogenous tobacco enzymes, which led to the accumulation of hederagenin-3-*O*-monoglucoside to levels far below those naturally occurring in *B. vulgaris* (Liu et al. 2019). When oleanolic acid monoglucoside was transiently produced in *N. benthamiana*, mainly nontoxic bidesmosidic saponins accumulated, suggesting that the toxic monoglucosides were further glycosylated and thereby detoxified by the plant (Khakimov et al. 2015). In addition, *P. xylostella* is a crucifer specialist and will not feed on *N. benthamiana*. To overcome these major obstacles, we metabolically engineered the saponin biosynthetic pathway by stable transformation in the wild crucifer *B. vulgaris* to test whether specific triterpenoid profiles confer preference to insect herbivory.

*B. vulgaris* occurs as 2 ecotypes that differ in both saponin profiles and insect resistance. The insect-susceptible P-type predominates in Eastern Europe, whereas the insect-resistant G-type is distributed from Central and Western Europe to Finland; however, both ecotypes co-occur in Denmark (Hauser et al. 2012; Agerbirk et al. 2015). To mimic the natural co-occurrence of both ecotypes in Denmark, we used preference feeding assays (choice test) rather than nonchoice conditions to better reflect the natural ecological conditions (Wang et al. 2017; Peláez et al. 2024).

We have previously hypothesized that variation in *LUP5* expression and LUP5 product specificity is a key driver of saponin-profile and insect herbivory differences between the 2 *B. vulgaris* ecotypes. This is based on quantitative trait locus analysis, insect feeding assays (choice as well as nonchoice) with saponin-treated leaf disks as well as through a mapping population generated from a cross of the G- and P-types, substantiated by in vitro enzyme bioactivity assay (Nielsen et al. 2010; Kuzina et al. 2011; Augustin et al. 2012; Khakimov et al. 2015; Liu et al. 2019; Günther et al. 2021). *LUP5* is 10-fold higher expressed in G-type as compared to P-type (Khakimov et al. 2015). In the insect-resistant G-type, LUP5 predominantly produces β-amyrin, directing metabolic flux toward oleanolic acid- and hederagenin-derived saponins that contribute to insect resistance (Augustin et al. 2012; Khakimov et al. 2015; Liu et al. 2019; Günther et al. 2021). In contrast, the P-type LUP5 enzyme, despite high sequence similarity, contains amino acid substitutions that shift its catalytic outcome toward α-amyrin, resulting in only minor production of β-amyrin-derived saponins (Günther et al. 2021).

To test this hypothesis in an in planta system, the G-type β-amyrin synthase *LUP5* was expressed in the insect-susceptible P-type *B. vulgaris*. In the otherwise insect-susceptible P-type, this resulted in up to a 95% reduction in leaf consumption (Fig. 2a). Accordingly, the G-type *LUP5* is a crucial determinant of insect preference in *B. vulgaris*. By combining gene transfer with ecological assays, our study provides a framework for linking specialized metabolism with ecological function in a nonmodel plant system.

To investigate which saponins contribute to feeding preference and how expression of G-type *LUP5* affects saponin composition, we tentatively identified and quantified saponins based on their fragmentation patterns and RTs in wild-type *B. vulgaris* and in G-type *LUP5*-expressing P- and G-type lines. Three hederagenin-derived monodesmosidic saponins (each containing 2 hexoses) and 1 oleanolic acid monoglucoside showed increased trends in G-type *LUP5*-expressing P-type plants compared with wild-type P-type plants (Fig. 3; Fig. 4, a and b). In the wild-type *B. vulgaris* G-type, hederagenin cellobioside is the predominant saponin, while only small amounts of the other 2 hederagenin-derived saponins were detected at RTs of 10.7 and 11.5 min (Fig. 6, Table S6). In contrast, in the *LUP5*-transformed P-type, the monodesmosidic hederagenin eluting at 11.5 min is more abundant than the other two (Fig. 4a). This variation highlights differences in saponin glycosylation patterns between P- and G-types, suggesting that these 3 hederagenin-derived monodesmosidic saponins, particularly the one detected at 11.5 min, may contribute to insect preference in the transformed P-type plants. Hederagenin 3-*O*-glucoside exhibits 7-fold stronger feeding reduction to diamondback moth (*P. xylostella*) and tobacco hornworm (*Manduca sexta*) compared to oleanolic acid 3-*O*-glucoside (Liu et al. 2019). This enhanced efficacy has been linked to C-23 hydroxylation in hederagenin, which causes the C-3 glucose moiety to adopt a different orientation relative to the sapogenin backbone compared to oleanolic acid (Cárdenas et al. 2019; Liu et al. 2019). Considering the bioactivity and the relatively low increase of oleanolic acid monoglucoside compared to hederagenin-derived monodesmosidic saponins in *LUP5*-transformed P-type suggests that there is a CYP72A552 ortholog in the P-type that effectively transforms oleanolic acid to hederagenin. Hederagenin is then glycosylated by different UGTs (UDP-glycosyltransferases) than in the G-type *B. vulgaris* to other linkage types than the characteristic 1→4 linkage cellobioside linkage that predominantly accumulates in the G-type.

Based on our findings, *LUP5* is the key determinant for insect resistance differences between G- and P-type *B. vulgaris*. Furthermore, 3 hederagenin-derived monodesmosidic saponins (each with 2 hexoses) may play a role in insect preference in *LUP5*-transformed P-type, particularly the most abundant saponin at RT 11.5 min. These findings offer mechanistic insight into plant–insect coevolution and establish a model for dissecting genotype–phenotype relationships in plant chemical defense. Future work comparing the insect resistance differences between the elucidated structures of these 3 hederagenin-derived monodesmosidic saponins could provide further insights into how glycosylation patterns affect saponin bioactivity. Glycoside linkage impacts cytotoxicity of saponins in vivo assays (Chwalek et al. 2006). However, the mode of action of how glycoside linkage affects insect resistance remains unknown. Unfortunately, due to their low concentration, isolating and purifying these compounds for bioactivity testing remains challenging. Future research should focus on identifying candidate glycosyltransferases that produce a variety of glycoside linkages with the aim of producing defense-related hederagenin-derived monodesmosidic saponins in planta or in other expression systems.

Interestingly, when *LUP5* expression was driven by the constitutive 35S promoter, *LUP5* was expressed at approximately 16-fold higher levels than in the best *pLUP5::LUP5* transgenic line and also exceeded expression levels in both wild-type P- and G-type plants (Fig. 2c). However, this high expression did not translate into a proportionally increased saponin production (Fig. 4, a and b, Fig. 6). In contrast, using the native G-type *LUP5* promoter not only resulted in approximately 2-fold higher transformation efficiency (Table 1) but also led to greater accumulation of hederagenin and its glycosides in the P-type background despite the overall much lower expression profile (Fig. 4, a and c). These findings indicate that gene expression strength alone does not determine metabolic output, but also choosing a promoter that facilitates coordinated expression with other necessary enzymes and available substrates is crucial.

We hypothesized that high expression of *LUP5* under the 35S promoter (Fig. 2c) leads to the accumulation of toxic intermediates or depletion of essential precursors. The strong constitutive 35S promoter is known to induce post-transcriptional gene silencing and may also influence the expression of neighboring genes at the integration site (Mlotshwa et al. 2010; Zhou et al. 2020). In *B. vulgaris*, G-type *LUP5* is expressed most strongly in leaves compared with roots and petioles (Khakimov et al. 2015). This tissue expression pattern is biologically important because it indicates where β-amyrin biosynthesis is naturally occurring and where defensive saponins are expected to be produced in response to insect attack. For this reason, in the present study, we compared promoter activity with a particular focus on leaf tissues. To better understand the observed differences in transformation efficiency, saponin and sapogenin accumulation, and *LUP5* expression levels between the native G-type *LUP5* and constitutive 35S promoters, we compared promoter activity and eGFP fluorescence intensity during plant regeneration and early growth. Microscopy observations support this interpretation: the 35S promoter was already active during shoot induction, whereas the native G-type *LUP5* promoter became detectable in leaves only 4 weeks after transfer to soil following root induction (Fig. 5). Such premature activation likely causes β-amyrin to accumulate before downstream pathway genes are expressed, disturbing metabolic balance through substrate depletion or feedback inhibition. By contrast, delayed activation under the native G-type *LUP5* promoter allows coordinated expression with downstream enzymes, promoting efficient accumulation of saponins that are nontoxic to the plant. To test whether the observed differences in transformation efficiency between the native G-type *LUP5* promoter and constitutive 35S promoter could be explained by tissue-specific expression, we examined promoter activity at the leaf tissue level. These data do not support a strict tissue-specific expression hypothesis, as both promoters were active across the leaf tissue (Fig. 5c).

Thus, temporal coordination of gene expression emerges as a key determinant of successful metabolic engineering. Although constitutive promoters such as 35S are widely used to achieve strong expression, their untimed activity can impose metabolic burdens and reduce transformation efficiency. Similar effects have been reported in other plant systems: in cotton (*Gossypium hirsutum*), continuous 35S activity throughout development caused unintended metabolic load (Sunilkumar et al. 2002), while in maize (*Zea mays*), the *ZmUbi1* promoter achieved higher transformation efficiency and better tissue-culture survival than 35S despite lower transcript levels (Beringer et al. 2017). These examples reinforce that expression timing, rather than transcriptional magnitude, is crucial for maintaining metabolic homeostasis and efficient pathway flux.

In conclusion, although the 35S promoter can drive high levels of transgene expression, its continuous activity may disrupt metabolic balance, decrease transformation efficiency, and induce pleiotropic effects on plant development. In contrast, the native G-type *LUP5* promoter confines expression to appropriate developmental stages, providing precise metabolic control and coordinated biosynthetic regulation. These results highlight that promoter choice governs metabolic outcomes, not merely through expression strength, but through temporal alignment with downstream biosynthetic processes, thereby ensuring efficiency in planta production of defense-related hederagenin-derived monodesmosidic saponins.

We expected CYP72A552 silencing to increase insect feeding, as hederagenin-derived saponins are more bioactive than those from oleanolic acid (Liu et al. 2019), but no effect was observed. Although hederagenin-derived saponins were decreased by ∼40% (Figure S3f), interpretation is complicated by 8 highly similar CYP72A paralogs (87% to 93% identity). These paralogs may cause off-target effects and cannot be distinguished by gene-specific RT-qPCR (Liu et al. 2019). Importantly, hederagenin cellobioside levels in G-type leaves remain well above the ED50 for *P. xylostella*, even after reduction, likely explaining the unchanged feeding behavior. Consistent with this, saponin cellobiosides correlate with insect resistance in a concentration-dependent manner, and hederagenin cellobioside levels in G-type leaves are ∼11-fold higher than the ED50 (Shinoda et al. 2002; Agerbirk et al. 2003; Kuzina et al. 2009; Liu et al. 2019).

## Conclusion

This study demonstrates that targeted metabolic engineering of triterpenoid saponins in the wild crucifer *B. vulgaris* provides a robust framework for dissecting the ecological roles of structurally diverse saponins and their contribution to insect feeding preferences mediated by specialized metabolism. Expression of the β-amyrin synthase G-type *LUP5* under its native G-type promoter was more effective than constitutive 35S-driven expression, resulting in higher transformation efficiency and increased accumulation of insect-deterring hederagenin-derived monodesmosidic saponins. These findings underscore the importance of promoter choice and precise transcriptional regulation in metabolic engineering strategies aimed at optimizing specialized metabolite production.

Our results establish stable metabolic engineering of saponin biosynthesis not only as a tool for biotechnological enhancement of resistance traits, but also as a powerful approach for elucidating the ecological functions of plant defense compounds in chemical ecology. In contrast to transient expression systems, which often result in unstable metabolite accumulation and additional modification by host enzymes, stable engineering enables consistent pathway gene expression and reliable functional assessment of metabolites.

We identify *LUP5* as a key determinant of insect feeding preference in *B. vulgaris* and demonstrate that 3 specific hederagenin-derived monodesmosidic saponins, each containing 2 hexose units, are associated with this defense. Taken together, this work introduces an application of metabolic engineering to unravel the ecological functions of plant specialized metabolites and highlights promising strategies for crop protection through fine-tuned manipulation of biosynthetic pathways.

## Materials and methods

### Plant and insect materials

Seeds of *B. vulgaris* G-type (glabrous, accession B44, Herbarium-code: CP0057358) and P-type (pubescent, accession B4, Herbarium-code: CP0057347) were provided by Associate Professor Niels Agerbirk (Agerbirk et al. 2021) and used for stable transformation. Both transformed and wild-type plants were cultivated in soil (Krukvaxtjord med lera och kisel, SW Horto AB, Hammenhog, Sweden) in a greenhouse maintained at 19 °C under a 16 h light/8 h dark photoperiod. Irradiance was supplemented with LED lamps whenever natural light intensity dropped below 250 µmol m^−2^ s^−1^, with full-spectrum sunlight available when curtains were open. Plants were fertigated on Mondays and Thursdays with a nutrient solution (electrical conductivity 2.2 mS cm^−1^; pH 5.8) prepared by dissolving 100 g L^−1^ of a compound fertilizer (Pioner Basis 13-2-23+3+ME; containing 13% N, 2% P_2_O_5_, 23% K_2_O, 3% MgO, plus micronutrients) together with 0.5 g L^−1^ of iron chelate (Pioner Fe-EDDHA 6%; 6% Fe) in water. *Brassica napus* plants were grown in the same greenhouse under identical conditions. *Plutella xylostella* (diamondback moth) eggs were obtained from Dr. Patrick Hughes (Boyce Thompson Institute, Ithaca, NY, United States). The laboratory colony was originally established at the New York State Agricultural Experiment Station (Geneva, NY, United States) in 1994. The insects were reared on *B. napus* at 20 °C under a 16 h light/8 h dark photoperiod in cages. Third instar *P. xylostella* larvae were used for the insect feeding assay.

### Plasmids construction

For stable transformation, the pJCV51 vector was employed to introduce the G-type *LUP5* coding sequence and to assess tissue-specific promoter activity (native *LUP5* and constitutive 35S), while pK7FWG2 was utilized for *CYP72A552* silencing (Karimi et al. 2002). Based on the genome sequence of *B. vulgaris* (Byrne et al. 2017), a 1,988 bp region upstream of the G-type *LUP5* was identified as its native promoter. This promoter fragment, along with the *LUP5* coding sequence, and the *CYP72A552* coding sequence were PCR-amplified from *B. vulgaris* G-type cDNA and genomic DNA using specific primers (Table S8 and S9). G-type *LUP5* expression was driven either by the 35S promoter or its native 1,988 bp promoter, while *CYP72A552* RNAi fragment was driven by the 35S promoter.

The pJCV51-p35S::LUP5, pJCV51-p35S::eGFP, and pK7FWG2-CYP72A552 RNAi plasmids were assembled using Gateway technology (Gateway BP Clonase II Enzyme mix, Thermo Fisher Scientific, 11789020; Gateway LR Clonase II Enzyme mix, Thermo Fisher Scientific, 11791020) (Table S8). pDONR207 was used as the entry clone vector for both constructs (Thermo Fisher Scientific, 117207-021). Subsequently, the pJCV51-pLUP5::LUP5 and pJCV51-pLUP5::eGFP plasmids were generated by replacing the 35S promoter in pJCV51-p35S::LUP5 or pJCV51-p35S::eGFP with the G-type *LUP5* promoter using the SalI sites. Finally, the plasmids were introduced into *Agrobacterium tumefaciens* strain AGL1 via electroporation (Hayta et al. 2021).

### Stable transformation in wild crucifer *B. vulgaris*

Seed sterilization was achieved by immersing seeds in 70% ethanol for 30 s, followed by a 12.5 min treatment in 3% sodium hypochlorite (Thermo Fisher Scientific) with 0.1% Tween20 (Bio-RAD, 1706531) and thorough rinsing with sterile water. Seeds were sown on germination medium in Magenta boxes and incubated in darkness for 6 to 8 days (Table S10).

Hypocotyl explants (∼3 mm) from 6- to 10-day-old seedlings were incubated for 15 min with *A. tumefaciens* strains (OD_600_ = 0.5 in 10 mM MgCl_2_, 100 µM acetosyringone) with gentle agitation and then co-cultivated in darkness for 3 days (Table S10). The pJCV51-pLUP5::LUP5 and pJCV51-35S::LUP5 constructs were transformed into both *B. vulgaris* ecotypes, while pJCV51-p35S::eGFP, pJCV51-pLUP5::eGFP, and pK7FWG2-CYP72A552 RNAi were introduced only into the G-type.

After rinsing in Milli-Q water containing 1 µL/mL timentin, the explants were transferred sequentially to callus induction (1 week), shoot induction (2 rounds, 2 weeks each), and root induction media (2 rounds, 2 weeks each) at 25 °C under a 16:8 h light–dark cycle (Table S10). Throughout these stages, the explants were maintained in a climate chamber at 25 °C under a 16:8 h light-dark cycle. Kanamycin (50 ng/mL) was used for selecting transformed explants, whereas wild-type explants were cultured without kanamycin supplementation in the medium.

Transgenic plants were confirmed by a combination of NPTII (neomycin phosphotransferase II) detection using an ELISA kit (Agdia, PSP 73000/0288) and fluorescence screening of 35S-driven RFP. Fluorescence was analyzed on a Leica M205FA fluorescence dissection microscope (Leica Microsystems). RFP and eGFP were imaged using the dsRed plant (excitation 546/10 nm; emission 600/40 nm) or ET GFP filters (excitation 470/40 nm; emission 525/50 nm), respectively. Subsequently, the confirmed plants were transferred to soil and grown in a greenhouse at 19 °C under a 16:8 h light–dark cycle.

### Choice insect feeding assay

Insect feeding preference was evaluated using at least 3 individual plants (biological replicates) per transgenic line. For each transgenic plant, 5 transgenic and 5 wild-type leaf discs (1.57 cm^2^ each) were alternately arranged in a petri dish (9.4 × 1.6 cm^2^) (Greiner BIO-ONE, 633180) with filter paper moistened with 2 mL of water. After a 3- to 5-h starvation period, 10 third-instar *P. xylostella* larvae were placed at the center of the plate, following a modified protocol from Liu et al. (2019). Leaf consumption was quantified when half of the wild-type leaves were consumed using ImageJ (approx. 7.5 h).

### Preparation of plant extracts and metabolite analysis

The extraction of saponins and sapogenins was adapted from Khakimov et al. (2016). *B. vulgaris* leaf samples were ground with liquid nitrogen using a mortar and pestle. For saponin detection, 100 mg of leaf powder was weighed into 1.5 mL Eppendorf tubes and extracted with 300 µL of 85% methanol (v/v) via sonication for 30 min, followed by centrifugation at 16,000 × *g* for 10 min. Supernatants (200 µL) were filtered through a 0.22-μm filter before LC-qTOF-ESI-MS/MS analysis.

To detect total and free sapogenins, 200 mg of leaf powder was weighed into 2 mL Eppendorf tubes and extracted with 600 µL of 85% methanol (v/v) at 100 °C while mixing at 1,400 × rpm in a thermomixer (Eppendorf, Denmark). The samples were cooled on ice and centrifuged at 16,000 × *g* for 3 min. After filtration through a 0.22-μm filter, 100 µL of supernatant was collected for free sapogenin detection via LC-qTOF-APCI-MS/MS. An additional 300 µL of supernatant was collected and dried under nitrogen flow. To cleave off sugar residues, samples were treated with 500 µL of 2 M hydrochloric acid at 100 °C while mixing at 1,400 × rpm for 1.5 h in a thermomixer (Eppendorf, Denmark). After cooling on ice, a double volume of ethyl acetate was added to the acid–water mixture, followed by vortexing and centrifugation at 3,500 × *g* for 5 min. The samples were extracted 3 times with ethyl acetate to collect sapogenins. To remove residual acid, an equal volume of Milli-Q water was added, and the samples were subsequently vortexed and centrifuged at 3,500 × *g* for 5 min. This washing step was repeated 3 times. The final extracts were dried under nitrogen flow, re-solubilized in 300 µL of 100% methanol, and filtered through a 0.22-μm filter before total sapogenin detection via LC-qTOF-APCI-MS/MS.

LC-qTOF-ESI-MS/MS was performed according to Liu et al. (2019). LC-qTOF-APCI-MS/MS was performed as described by Patel et al. (2020). Untargeted metabolite data were analyzed using XCMS (Tautenhahn et al. 2012), and targeted saponin and sapogenin analyses were conducted with DataAnalysis software (v.4.3; Bruker, Bremen, Germany) following Khakimov et al. (2016). Hederagenin cellobioside and oleanolic acid cellobioside standards were used for saponin identification. Lupeol, β-amyrin, oleanolic acid, and hederagenin standards were employed for sapogenin identification.

### Gene expression analysis

Total RNA was extracted using the Spectrum Plant Total RNA Kit (Sigma-Aldrich, STRN50-1KT) and then synthesized to cDNA by iScript cDNA Synthesis Kit (Bio-Rad, 1708891). Relative expression of *LUP5* was quantified via RT-qPCR using Kapa SYBR Fast kit (Merck-Sigma, KK4607) (Khakimov et al. 2015). *Tubulin* (GenBank accession no. EU555191) was used as a reference gene, and *LUP5* expression levels were normalized to the wild-type P-type control (Liu et al. 2016). Primer sequences are provided in Table S8 (Khakimov et al. 2015).

### Accession numbers

Sequence data from this article can be found in the GenBank/EMBL data libraries under accession numbers MH252567 to MH252574 (*CYP72As*) and KP784690.1 (G-type *LUP5*).

## Supplementary Material

kiag291_Supplementary_Data

## Data Availability

The data underlying this article are available in the article and in its online supplementary material.
