# Supplementary material for: Metabolic engineering reveals LUP5 as a determinant of saponin composition and insect feeding preference in Barbarea vulgaris
Source: Plant Physiol. 2026 May 22;201(3):kiag291. doi: 10.1093/plphys/kiag291 (PMC13353108; doi:10.1093/plphys/kiag291)
Supplement: kiag291_Supplementary_Data [file kiag291_supplementary_data.pdf]

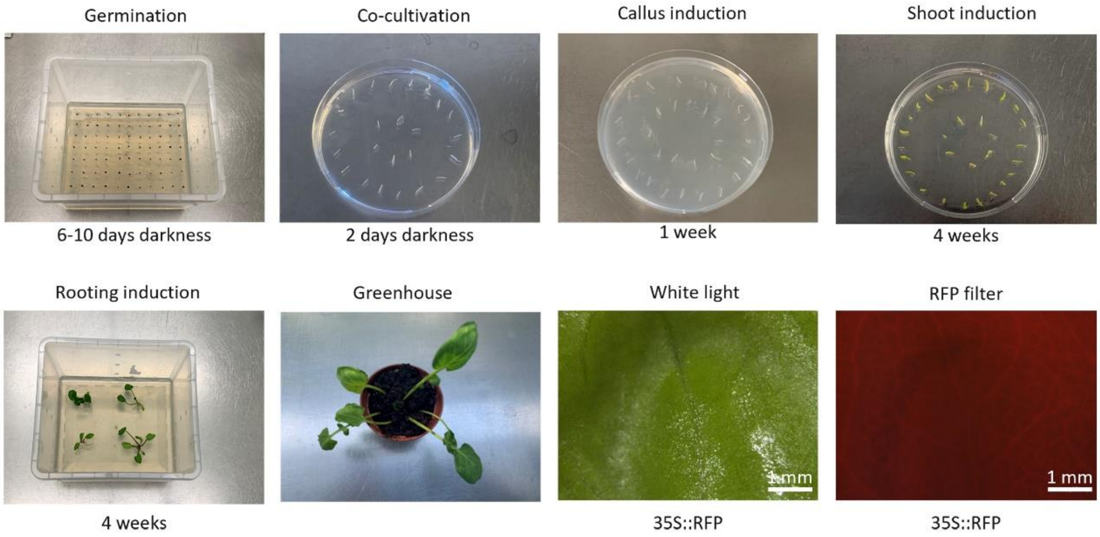

Supplementary Figure S1. Agrobacterium-mediated stable plant transformation process. To establish a causal link between saponin composition and insect feeding preference, a stable transformation and regeneration system was developed for both ecotypes of *B. vulgaris*. Transgenic plants were obtained through seed germination, *Agrobacterium* co-cultivation, callus induction, shoot and root regeneration, and subsequent transfer to soil in the greenhouse. Transgenic plants were selected by ELISA detection of the NPTII gene product and by fluorescence screening of 35S-driven RFP.

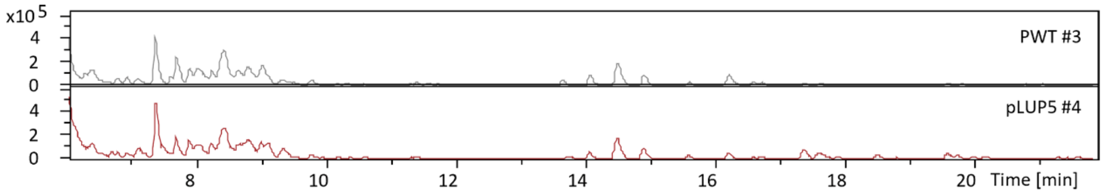

Supplementary Figure S2. Untargeted metabolite analysis was not sufficiently sensitive to detect saponin differences in this study. No clear differences in base peak chromatogram intensity were observed between wild-type P-type and *LUP5*-transformed P-type *B. vulgaris*, even for the four oleanolic acid– and hederagenin-derived saponins that were increased in transgenic plants. In contrast, targeted LC–MS analysis revealed increased levels of glycosylated oleanolic acid (eluting at 9.5 min) and hederagenin derivatives (eluting at 10.7, 11.5, and 12.3 min) in P-type plants expressing G-type *LUP5*, as shown in Figures 3A and 4. PWT #3, wildtype P-type *B.*

vulgaris line 3; pLUP5 #4, pLUP5::LUP5 transformed P-type *B. vulgaris* line 4.

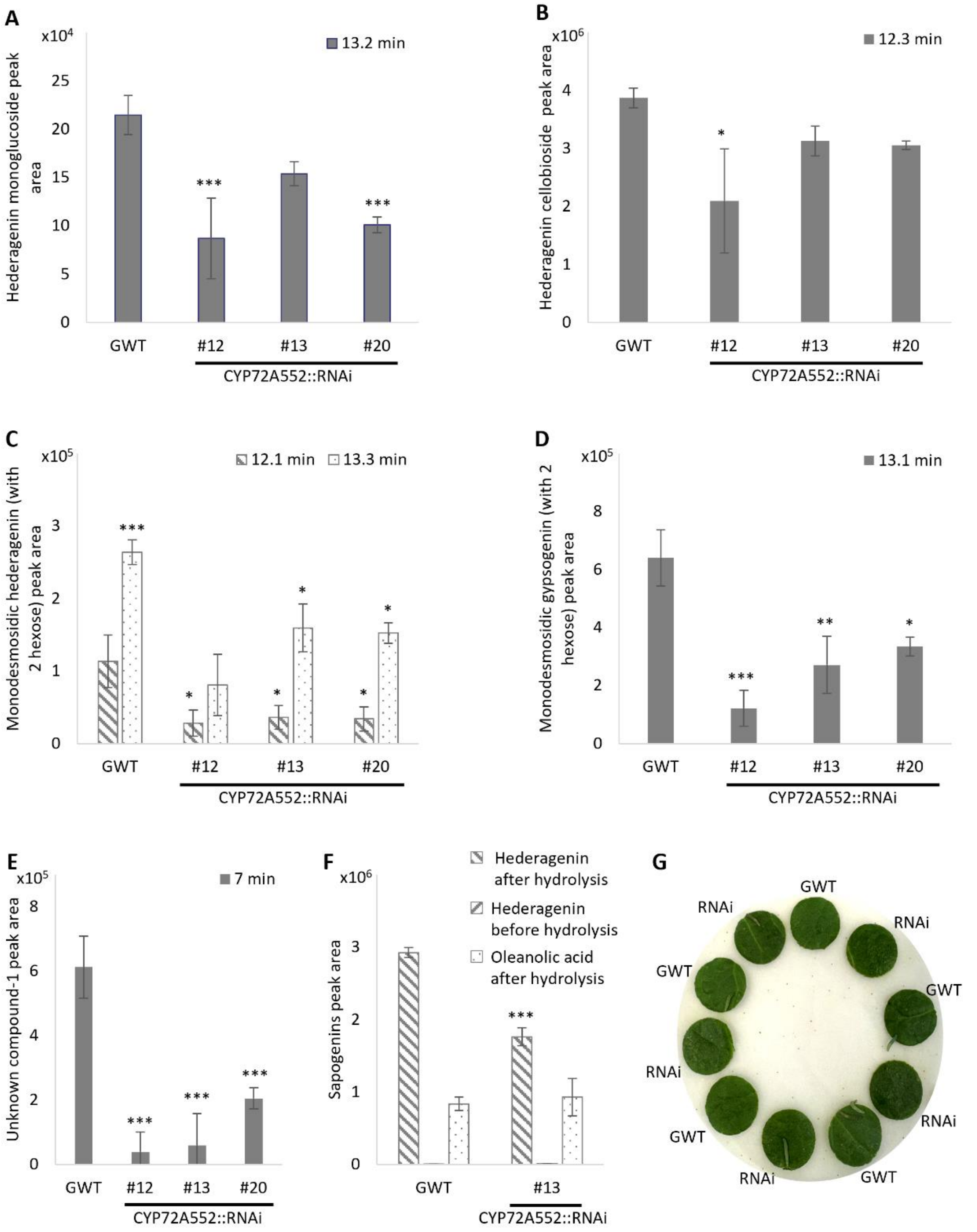

Supplementary Figure S3. Hederagenin and its derived saponins showed significantly reduced levels in G-type *B. vulgaris* after *CYP72A552* silencing. To assess the effect of *CYP72A552* silencing on insect feeding preference and on saponin and sapogenin levels, we performed choice feeding assays and tentatively identified and quantified saponins in leaf extracts from wild-type and *CYP72A552* silenced G-type plants using LC–MS/MS. A) Hederagenin monoglucoside peak area (EIC: m/z = 679.41 ± 0.2). GWT,

wildtype P-type *B. vulgaris*; *CYP72A552*::RNAi (RNAi), *CYP72A552* silenced G-type *B. vulgaris*. B) Hederagenin cellobioside peak area (EIC: *m/z* = 841.46 ± 0.2). C) Monodesmosidic hederagenin (with two hexoses) peak area (EIC: *m/z* = 841.46 ± 0.2). D) Monodesmosidic gypsogenin (with two hexoses) peak area (EIC: m/z = 839.44 ± 0.2). E) unknown compound-1 peak area (EIC: *m/z* = 901.24 ± 0.2). MS/MS analysis indicated that the unknown saponin contains a sapogenin of 457 m/z with one hexose and two methylpentoses. F) Sapogenin peak area. To evaluate the effect on sapogenin levels, *CYP72A552*-silenced line #13 was chosen as a representative line of the three best performing lines and analyzed across three vegetative clones. Hederagenin was detectable both before and after acid hydrolysis (EIC, *m/z* 437.34 ± 0.2), whereas oleanolic acid was detectable only after acid hydrolysis (EIC, *m/z* 439.36 ± 0.2). Acid hydrolysis with hydrochloric acid (HCL) was used to remove sugar moieties from saponins, thereby releasing the sapogenin backbone for detection. Without hydrolysis, sapogenins were detected only when present in the free (non-glycosylated) form. G) Example of the insect choice feeding assay, and the resulting leaf condition after 7.5 h insect exposure. Numbers below the bars indicate independent transgenic lines. Error bars represent the standard deviation of the mean from three individual plants. Statistical significance was assessed using ANOVA with wildtype G-type as the control. Asterisks indicate significant differences * (p < 0.05), ** (p < 0.01), and *** (p < 0.005).

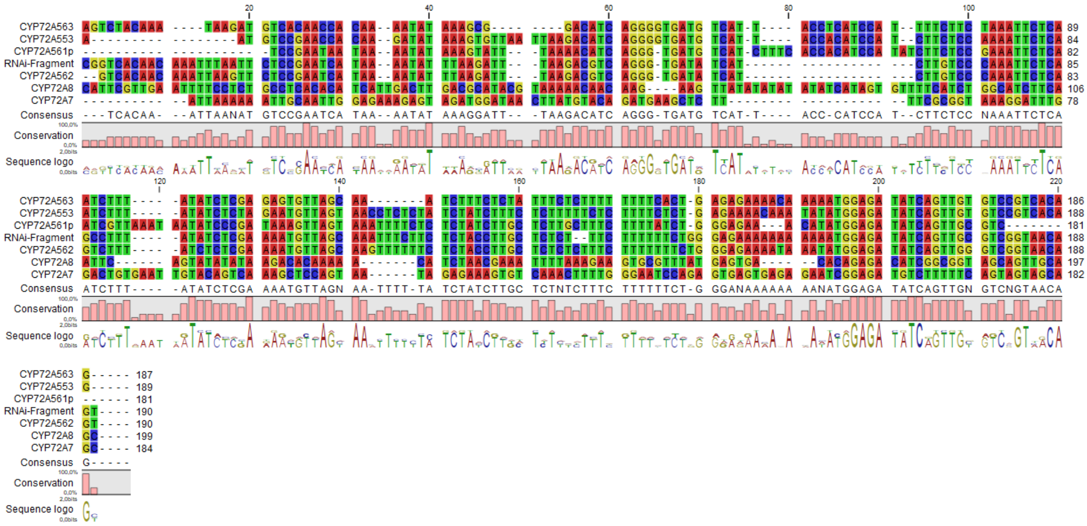

Supplementary Figure S4. Nucleotide sequence alignment between the RNAi fragment and eight tandemly repeated *CYP72A* genes in *B. vulgaris*. Accession numbers of the eight *CYP72A* genes range from MH252567 to MH252574.

Supplementary Table S1. Feature level changed by *LUP5* expression in P-type *Barbarea vulgaris.*

Feature category	pLUP5	p35S	Both promoter lines
Total detected features	9500	9500	9500
Features with higher abundance	2143	2544	1655
Features with lower abundance	1758	1357	869

Supplementary Table S2. Saponins detected in P-type *B. vulgaris*.

Sapogenin	Saponin fragment	Sugar moieties	Formic acid	Fragmentation pattern in Mass Spectra	EIC±0.2	Retention time (min)		
457	457,619,781,943,989	3 x hexose	Yes	[M_{989}-46-162-162-162=457]	989.5404	8.6	8.9	
447	447,609,755,901	1 x hexose; 2 x methylpentose	No	[M_{901}-146-146-162=447]	901.2429	6.3		
473	473,635,797,959,1005	3 x hexose	Yes	[M_{1005}-46-162-162-162=473]	1005.5337	8.1	8.4	
473	473,635,797,843	2 x hexose	Yes	[M_{843}-46-162-162=473]	843.4798	8.6		
471	471,633,795,841	2 x hexose	Yes	[M_{841}-46-162-162=471]	841.4591	10.7	11.5	12.3
455	455,617	1 x hexose	No	[M_{617}-162=455]	617.4059	9.5		

Note: EIC, extract ion chromatogram.

Supplementary Table S3. Saponins detected in G-type *B. vulgaris*.

Sapog genin	Saponin fragment	Sugar moieties	Formic acid	Fragmentation pattern in Mass Spectra	EIC±0.2	Retention time (min)				
457	457,619,781,943,989	3 x hexose	Yes	[M_{989}-46-162-162-162=457]	989.5404	8.6	8.9	9.1		
447	447,609,771,917,1079, 1225	3 x hexose ; 2 x methylpentose	No	[M_{1225}-146-162-146-162-162=447]	1225.348	5.3				
447	447,609,755,901	1 x hexose; 2 x methylpentose	No	[M_{901}-146-146-162=447]	901.2429	7.0				
649	649,811,973,1019	2 x hexose	Yes	[M_{1019}-46-162-162=649]	1019.5076	7.9				
649	649,811,857	1 x hexose	Yes	[M_{857}-46-162=649]	857.4543	8.4				
469	469,631,793,839	2 x hexose	Yes	[M_{839}-46-162-162=469]	839.4409	13.1				
473	473,635,797,959,1005	3 x hexose	Yes	[M_{1005}-46-162-162-162=473]	1005.5337	8.4				
473	473,635,797,843	2 x hexose	Yes	[M_{843}-46-162-162=473]	843.4798	8.0				
471	471,633,795,841	2 x hexose	Yes	[M_{841}-46-162-162=471]	841.4591	10.7	11.5	12.1	12.3	13.3
471	471,633,795	2 x hexose	No	[M_{795}-162-162=471]	795.4536	9.5	11.7			
471	471,603,749,795	1 x pentos; 1 x methylpentose	Yes	[M_{795}-46-146-132=471]	795.4536	13.8				
471	471,633,679	1 x hexose	Yes	[M_{679}-46-162=471]	679.4063	13.2				
471	471,633,795,957,1003	3 x hexose	Yes	[M_{1003}-46-162-162-162=471]	1003.5115	8.5	9.3			
455	455,617	1 x hexose	No	[M_{617}-162=455]	617.4059	15.9				
455	455,617,779,941	3 x hexose	No	[M_{941}-162-162-162=455]	941.5115	10.2				
455	455,617,779,941,987	3 x hexose	Yes	[M_{987}-46-162-162-162=455]	987.517	10.7				
455	455,617,779	2 x hexose	No	[M_{779}-162-162=455]	779.4587	11.0	11.5	14.2		
455	455,617,779,825	2 x hexose	Yes	[M_{825}-46-162-162=455]	825.4642	11.3	13,3	14.5		

Note: EIC, extract ion chromatogram.

Supplementary Table S4. Non significantly peak area changed saponins in *LUP5* transformed P-type *B. vulgaris* compared to wildtype P-type.

Fragments	Sugar moieties	EIC ± 0.2	Retention time (min)	Peak area						
				PWT	p35S::LUP5-1	p35S::LUP5-2	p35S::LUP5-43	pLUP5::LUP5-2	pLUP5::LUP5-4	pLUP5::LUP5-6
457,619,781, 943,989	3 x hexose	989.5404	8.6	104397 ± 14484	76740 ± 6720	78388 ± 5652	116101 ± 7003	105183 ± 12223	96111 ± 4826	96408 ± 6982
457,619,781, 943,989	3 x hexose	989.5404	8.9	103949 ± 22256	160286 ± 11490	164287 ± 6733	90535 ± 2995	93870 ± 16808	120504 ± 23440	112588 ± 6319
447,609,755, 901	1 x hexose + 2 x methylpentose	901.2429	6.3	514487 ± 15989	515685 ± 14393	681218 ± 36231	456469 ± 39764	460209 ± 82384	491958 ± 19291	399510 ± 15858
473,635,797, 843	2 x hexose	843.4798	8.6	379938 ± 129721	767152 ± 82948	793761 ± 38062	373927 ± 25068	283426 ± 26935	497737 ± 137792	413195 ± 21207
473,635,797, 959,1005	3 x hexose	1005.5337	8.1	731408 ± 68292	700227 ± 1622	678699 ± 28074	647943 ± 3678	615053 ± 23641	618340 ± 7003	584503 ± 9686
473,635,797, 959,1005	3 x hexose	1005.5337	8.4	248947 ± 33719	309143 ± 5811	328133 ± 1430	235073 ± 10365	213887 ± 21875	256559 ± 16436	209736 ± 7098

Note: EIC, extract ion chromatogram; PWT, wildtype P-type B. vulgaris; p35S::LUP5, p35S::LUP5 transformed P-type B. vulgaris.

Supplementary Table S5. Identification and relative quantification of total and free sapogenins in *B. vulgaris*.

Sapogenins	EIC±0.2	Retention time (min)	PWT	p35S::LUP5-2	pLUP5::LUP5-4	GWT	CYP72A552::RNAi-13
Total lupeol	409.3851	31.1	N/A	N/A	N/A	N/A	N/A
Free lupeol	409.3851	31.1	N/A	N/A	N/A	N/A	N/A
Total β-amyrin	409.3851	32.5	N/A	N/A	N/A	N/A	N/A
Free β-amyrin	409.3851	32.5	N/A	N/A	N/A	N/A	N/A
Total oleanolic acid	439.3593	21.5	20884 ± 3963	45589 ± 6970	53763 ± 5506	837805 ± 94973	930214 ± 259536
Free oleanolic acid	439.3593	21.5	N/A	N/A	N/A	7694 ± 2187	12102 ± 3978
Total hederagenin	437.3425	16.7	37387 ± 13280	119157 ± 28689	316704 ± 75506	2923104 ± 69572	1763484 ± 124066
Free hederagenin	437.3425	16.7	N/A	N/A	N/A	N/A	N/A

Note: EIC, extract ion chromatogram. Lupeol and β-amyrin remained undetectable in G-type *B. vulgaris*.

Supplementary Table S6 Non significantly peak area changed saponins in LUP5 transformed G-type *B. vulgaris* compared to wildtype G-type.

Fragments	Sugar moieties	EIC ± 0.2	Retention time (min)	Peak area						
				GWT	p35S::LUP5-3	p35S::LUP5-4	p35S::LUP5-8	pLUP5::LUP5-1	pLUP5::LUP5-4	pLUP5::LUP5-19
457,619,781, 943,989	3 x hexose	989.5404	8.6 min	20482 ± 2118	38987 ± 1098	51472 ± 15255	21690 ± 3768	19634 ± 865	20222 ± 1111	31016 ± 5656
457,619,781, 943,989	3 x hexose	989.5404	8.9 min	14821 ± 1198	18031 ± 2051	17787 ± 1628	12320 ± 1638	18273 ± 2536	18420 ± 1669	21301 ± 3786
457,619,781, 943,989	3 x hexose	989.5404	9.1 min	38031 ± 3104	52061 ± 5462	65168 ± 12087	35694 ± 2021	48842 ± 4730	49031 ± 4808	51257 ± 2596
447,609,771, 917,1079,1225	3 x hexose 2 x methylpentose	1225.3480	5.3 min	13663 ± 2246	18448 ± 3368	22842 ± 4336	15106 ± 3645	25363 ± 6389	25538 ± 6666	20553 ± 5429
447,609,755, 901	1 x hexose 2 x methylpentose	901.2429	7.0 min	120237 ± 16382	234354 ± 60723	265639 ± 33723	153354 ± 46292	103209 ± 18904	158676 ± 26195	69202 ± 27745
649,811,973, 1019	2 x hexose	1019.5076	7.9 min	21216 ± 6337	46666 ± 8855	55221 ± 9747	32606 ± 7696	24344 ± 7109	19506 ± 5435	24221 ± 3354
649,811,857	1 x hexose	857.4543	8.4 min	12711 ± 5812	54358 ± 10282	39625 ± 11515	11513 ± 1677	8841 ± 1206	6367 ± 1110	12549 ± 4138
469,631,793, 839.4409	2 x hexose	839.4409	13.1 min	184062 ± 9520	177262 ± 6220	188763 ± 36759	162025 ± 11433	163858 ± 9362	158627 ± 16412	169893 ± 1543
473,635,797, 959,1005	3 x hexose	1005.5337	8.4 min	26331 ± 1839	30415 ± 1107	32803 ± 6153	18898 ± 1143	22765 ± 1235	23008 ± 3232	29006 ± 6414
473,635,797, 843.4798	2 x hexose	843.4798	8.0 min	17071 ± 5223	10868 ± 3119	14373 ± 6021	10972 ± 1123	21960 ± 1914	25028 ± 5756	11849 ± 1801
471,633,795, 841	2 x hexose	841.4591	12.3 min	100456 ± 5418	106334 ± 12545	110089 ± 8301	95604 ± 2647	107654 ± 11391	102554 ± 10010	96034 ± 5295
471,633,795, 841	2 x hexose	841.4591	10.7 min	33658 ± 5344	37416 ± 8271	38169 ± 6400	31962 ± 9915	47190 ± 3606	45222 ± 2232	27295 ± 7916
471,633,795, 841	2 x hexose	841.4591	11.5 min	25386 ± 3902	24225 ± 3487	29513 ± 3269	18135 ± 1956	32274 ± 3485	28554 ± 3328	22019 ± 5539
471,633,795, 841	2 x hexose	841.4591	12.1 min	47036 ± 16885	32613 ± 17910	29637 ± 9302	19750 ± 14769	47282 ± 7261	45142 ± 2657	25284 ± 6002
471,633,795, 841	2 x hexose	841.4591	13.3 min	88619 ± 2761	90928 ± 7073	95672 ± 18388	90196 ± 5713	86544 ± 4723	83258 ± 8292	92525 ± 5391
471,633,795	2 x hexose	795.4536	9.5 min	5117 ± 699	6368 ± 987	6909 ± 2907	6047 ± 640	9009 ± 1110	7197 ± 861	8855 ± 1485
471,633,795	2 x hexose	795.4536	11.7 min	218249 ± 23129	216482 ± 35533	230016 ± 16668	210209 ± 23914	309077 ± 21009	301037 ± 23688	200395 ± 82158
471,603,749, 795	1 x pentos 1 x methylpentose	795.4536	13.8 min	17769 ± 2552	22284 ± 3550	65568 ± 57745	19566 ± 2099	25555 ± 1657	26195 ± 1627	18956 ± 4763
471,633,679	1 x hexose	679.4063	13.2 min	212610 ± 36755	233137 ± 4815	207951 ± 34240	207624 ± 16534	167168 ± 11718	179878 ± 11956	208232 ± 22129
471,633,795, 957,1003	3 x hexose	1003.5115	9.3 min	11634 ± 2468	11052 ± 1548	10676 ± 2002	20561 ± 6247	18977 ± 2852	16839 ± 3467	23814 ± 3476
455,617	1 x hexose	617.4059	15.9 min	10294 ± 1439	13237 ± 2571	13947 ± 1427	15815 ± 829	17539 ± 283	17940 ± 1969	12755 ± 3767
455,617,779, 941	3 x hexose	941.5115	10.2 min	2146 ± 144	2830 ± 851	3396 ± 249	2760 ± 899	3733 ± 575	3265 ± 921	2592 ± 375
455,617,779	2 x hexose	779.4587	11.5 min	5830 ± 286	7906 ± 539	5088 ± 2615	7406 ± 635	5930 ± 366	8004 ± 812	6783 ± 1732
455,617,779	2 x hexose	779.4587	14.2 min	2662 ± 1048	4302 ± 1240	5675 ± 814	2779 ± 669	5970 ± 1384	5353 ± 735	3217 ± 1321
455,617,779, 825	2 x hexose	825.4642	11.3 min	9091 ± 1213	7393 ± 793	10092 ± 3281	9520 ± 1552	10141 ± 1139	9067 ± 2291	9807 ± 1897
455,617,779, 825	2 x hexose	825.4642	13,3 min	71245 ± 20814	92414 ± 31302	113829 ± 25789	98847 ± 34763	176841 ± 21237	178891 ± 7069	89426 ± 54408
455,617,779, 825	2 x hexose	825.4642	14.5 min	243257 ± 17534	274932 ± 25755	308652 ± 55083	298511 ± 15815	308259 ± 6512	499973 ± 154132	303445 ± 31768

Note: EIC, extract ion chromatogram; GWT, wildtype G-type *B. vulgaris*; p35S::LUP5, p35S::LUP5 transformed G-type *B. vulgaris*. Hederagenin cellbioside (EIC: *m/z* 841.4591± 0.2, retention time 12.3 min) was quantified by after diluting sample 60 times

Supplementary Table S7. Non significantly peak area changed saponins in CYP72A552 RNAi G-type *B. vulgaris* compared to wildtype G-type.

Fragments	Sugar moieties	EIC ± 0.2	Retention time (min)	Peak area			
				GWT	CYP72A552::RNAi-12	CYP72A552::RNAi-13	CYP72A552::RNAi-20
457,619,781,943,989	3 x hexose	989.5404	8.6	59779 ± 11070	22819 ± 12186	50571 ± 16040	114054 ± 20836
457,619,781,943,989	3 x hexose	989.5404	8.9	138182 ± 17852	73616 ± 40884	128750 ± 25492	191028 ± 39924
457,619,781,943,989	3 x hexose	989.5404	9.1	114945 ± 21459	58662 ± 32186	103841 ± 16971	193364 ± 49342
447,609,771,917,1079,1225	3 x hexose<br>2 x methylpentose	1225.3480	5.3	46154 ± 7366	21556 ± 14434	71688 ± 59579	113983 ± 69871
649,811,973,1019	2 x hexose	1019.5076	7.9	135335 ± 19808	55449 ± 29797	88059 ± 16763	65890 ± 20234
649,811,857	1 x hexose	857.4543	8.4	77952 ± 10900	25061 ± 12578	50488 ± 8478	49935 ± 17760
473,635,797,959,1005	3 x hexose	1005.5337	8.4	113195 ± 20812	29081 ± 13620	56670 ± 9168	145066 ± 50308
473,635,797,843.4798	2 x hexose	843.4798	8.0	31446 ± 7784	12140 ± 5535	18386 ± 2755	19709 ± 5620
471,633,795,841	2 x hexose	841.4591	10.7	136220 ± 38770	76534 ± 45911	99014 ± 25908	82659 ± 26899
471,633,795,841	2 x hexose	841.4591	11.5	35229 ± 5872	11376 ± 3350	18245 ± 5585	25617 ± 5052
471,633,795	2 x hexose	795.4536	9.5	90519 ± 8592	29836 ± 11870	58144 ± 15541	66536 ± 14249
471,633,795	2 x hexose	795.4536	11.7	728536 ± 151774	470860 ± 306512	665595 ± 140211	582805 ± 161723
471,603,749,795	1 x pentos<br>1 x methylpentose	795.4536	13.8	550036 ± 42464	380767 ± 129601	494529 ± 57332	285280 ± 150650
471,633,795,957,1003	3 x hexose	1003.5115	8.5	60301 ± 7106	41708 ± 24516	60618 ± 18649	36844 ± 8278
471,633,795,957,1003	3 x hexose	1003.5115	9.3	47445 ± 9326	13747 ± 6648	32703 ± 11770	87323 ± 26478
455,617	1 x hexose	617.4059	15.9	143159 ± 29068	132769 ± 98284	175934 ± 61931	52051 ± 22730
455,617,779,941	3 x hexose	941.5115	10.2	22899 ± 4464	14295 ± 7815	25146 ± 5922	23357 ± 5447
455,617,779,941,987	3 x hexose	987.5170	10.7	21236 ± 2477	16389 ± 7742	27686 ± 5895	38374 ± 8331
455,617,779	2 x hexose	779.4587	11.0	99602 ± 24999	62016 ± 54628	68706 ± 46239	86853 ± 28604
455,617,779	2 x hexose	779.4587	11.5	71824 ± 16651	52094 ± 28782	68902 ± 18317	75586 ± 28924
455,617,779	2 x hexose	779.4587	14.2	29037 ± 6717	15961 ± 11135	23933 ± 1307	19142 ± 2578
455,617,779,825	2 x hexose	825.4642	11.3	29293 ± 1026	20550 ± 10861	26234 ± 4644	30276 ± 7026
455,617,779,825	2 x hexose	825.4642	13.3	321419 ± 118351	201969 ± 167251	291024 ± 109151	266926 ± 75364
455,617,779,825	2 x hexose	825.4642	14.5	2161276 ± 255225	1722183 ± 777331	2351852 ± 301176	1599796 ± 531805

Note: EIC, extract ion chromatogram; GWT, wildtype P-type *B. vulgaris*; CYP72A552::RNAi, *CYP72A552* silenced G-type *B. vulgaris*.

Supplementary Table S8. Primers used in this study.

	Oligo name	Sequence 5'-3'
Amplication	LUP5_Fw	ATGTGGAGGTTGAAGTTAGGAGAGG
	LUP5_Rv	GCTGCGTTCGTCACTCATAAACATTAA
	LUP5_Promoter_Fw	GGATACGGATACGGATACGGAT
	LUP5_Promoter_Rv	GTTGTACTCTACATATAGATCATCATT
	eGFP-Fw	ATGGTGAGCAAGGGCGA
	eGFP-Rv	TTACTTGTACAGCTCGTCCATG
Gateway	LUP5_attB1_Fw	GGGGACAAGTTTGTACAAAAAAGCAGGCTATGTGGAGGTTGAAGTTAGGAGAGG
	LUP5_attB2_Rv	GCTGCGTTCGTCACTCATAAACATTAAACCCAGCTTTCTTGTACAAAGTGGTCCCC
	CYP72A552_attB1_Fw	GGGGACAAGTTTGTACAAAAAAGCAGGCTCGGTCACAACAAATTTAATTCTCCGAATC
	CYP72A552_attB2_Rv	GAGATATCAGTTGCGTCGGTAACAGTACCCAGCTTTCTTGTACAAAGTGGTCCCC
	eGFP_attB1_Fw	GGGGACAAGTTTGTACAAAAAAGCAGGCTATGGTGAGCAAGGGCGA
	eGFP_attB2_Rv	GGGGACCACTTTGTACAAGAAAGCTGGGTTTACTTGTACAGCTCGTCCATG
RT-qPCR	Tubulin qPCR Fw	GGAGATGTTTAGGCGTGTG
	Tubulin qPCR Rv	GCGTCTTGGTATTGCTGGT
	LUP5 qPCR Fw	CTGAGCGTGAGTACGTGGAA
	LUP5 qPCR Rv	GATCTGGATAGAGTTGCTGGA

Supplementary Table S9. Nucleotide sequence of the G-type *LUP5* promoter.

GGATACGGATACGGATACGGATACGGATAGTAGAATAACGGATAATACGAATACGGATACAGATACGGATATCCTGAAAAATCCGGATATCCGTATCCGT
AACAGGGCTATTACTGACCCCACCCGTCCGGCAGTAGAGTCCGTCTACGTCCTGCGTCCCCTCATTCATTGTACCTATGACAAAGACGCATAGTTATTA
GAGCTACACCGCCGCGACTTTGCTGCTCCTCCACTCATACATTATCATAGCTAAGTTCTCAAAAAGATTCAATCAAAGCCCTTCTCTTTGTATGGGACCA
TTTTACACGTGATTATGTATCAACGTTACTTGCTCATTGACAAAATTGAGTTAGGTCTCTCCTCTTTTGCTCTATTTTACTTCTATATTTGTCAATTTACTCT
ACTATGCATCTAATTATGACGGTACATGAAGTCCTTATGGTTAGGTTTGCCACCGGTTGTGATTACAAATTTATTAGAATTTATCCCATGGTTAGAGAATT
TATAAGTTTTTTTTCATTAGTTATATTATAATATTTTCTAAGTATAAGGTTGAATATGAGTATTAAAAAAAAAAAAGAGAGAGAACTAACAATGGATAAAATA
TCATATGACTGAAATTTTTTTATACATTCCGTAAGGAACTAAGGATGATTATTAGTATAAAGATTCATTGCATCAAACAATTATGGTTAAGTAGTTAATTTA
CATCAATTTGGTATAATCAACATCCAATTTAAAAAATACGCGTCACACAAAGATTAAACCCAAATACACATGTGATAAAGAGAAGAACAAAGATGACCA
TCAATTACGCTGGTTGTCTTTGCATGAATGGAACACCTAATCGCGGGAAACTTTTTGAATCAGCGACTCAAACATACTTCGTTTCTACATAGGTGTCATT
CAATATAATTACATATGTAGGTAGCTAGTTTTAACGGACTGATCATAATTTTTGTAGGTTTTTTTTTTTTTAATAACTAAAAGGTTTTAGGTCTAAGCCTGTA
CATCCTTTTAGACCCAGGACCAAAGCAGGTTATATGGTCTCGTCCCATGAGCCACTCGAACCAACGACCTCTAACCTTTGCCTGAGATATTTACCAGTT
GAGTTATTAGCTCTTGGTTAATTTTTATAGGTTTAAATCAAATGTATCTCATAATGATCGATATAACAAACAATAAACAAAAAAAGTTTGAAGGTGAATTT
TGCACCCAAAAAAAAAAAAAAACTTTTTATTATCTTTTTCATTTTTTTTATAAATCACGGGAGTTCTAAACCTTTGACCCGTAATCTTTTTCGGTATCCGGA
ACCAGCCTTTACTCTATTTCTATTTTAAAAATGTCTCTCAGTACCAATGGAGCCGGGTTTGAACCCGTGAAACAAAAATATTTATTTATTATTTATTATTATA
TTTTGGAAAGAACGTAAAAGTTATTTCAACGAAAAAAAATGTGGGAATATAAATAAAAAAAAAAATTCAATATAATATTAAATTATTTACCAAACTGTACTC
TTATAAAGTTATTTGATTCGTCGTTACCTCTATATAAGCGTACGTACATGAAACTCTTCCAAAGTATTGGTGATAAAGGCAAACATCGACCCAGCACAAG
TACTCTCTCTTTCTTTCTCTTATCTCACTTTTCCTCTGTTTCACATATATGTATTCGTTACATGAGGGCTCTTATAATATATAAAGACATGCATATAAAATCATA
TATATATATATATATGTATGTAAATATAGTGTTCTATATATAATGTTAATCTATGTATACGGTATACCGTATGAATGTACACTCACTACATCATATATCATCATGCG
GTTATGAAATTCTCAATTCAGTGAGTATAATTTGGACATATACGTATAGATAGCGTCTAAATTTTTGTAATTTTTTAAAAAAAAAATAAATGAATGACGGTT
TGAAACGAACATAACAATAACATATATCGATATTTTGTTGTACTCTACATATAGATCATCATT

Supplementary Table S10. Media for tissue culture.

	Germination		Co-cultivation		Callus induction		Shoot induction		Root induction	
Media (800 mL)	MS (M0222)	3.52 g	MS (M0222)	3.52 g	Gamborg B5	3.16 g	Gamborg B5	3.16 g	Gamborg B5	3.16 g
	Sucrose (1%)	8 g	Sucrose (1%)	8 g	MES	400 mg	MES	400 mg	MES	400 mg
	pH 5.8 (with KOH)		pH 5.8 (with KOH)		Glucose	16 g	Glucose	16 g	Glucose	16 g
	Agar (0.8%)	6.4 g	Agar (0.8%)	6.4 g	pH 5.8 (with KOH)		pH 5.8 (with KOH)		pH 5.8 (with KOH)	
					Phytagel	2.4 g	Phytagel	2.4 g	Phytagel	2.4 g
Adding before using (800 mL)			AS	800 μL	Kinetin	80 μl	Zeatin	800 μl	NAA	108 μl
					2,4-D	400 μl	NAA	80 μl	IAA	50 μl
					Timentin	800 μl	Timentin	800 μl	Timentin	800 μl
					Kanamycin	800 μl	Kanamycin	800 μl	Kanamycin	800 μl

Note: Chemical information: MS, Murashige & Skoog medium including vitamins (Duchefa, M0222.0050); Gamborg B5 (Duchefa, G0210.0050). Stocks concentration: Kanamycin: 50 mg/mL, Timentin: 200 mg/mL, Kinetin: 1 mg/mL, Zetin: 1 mg/mL, NAA (naphthalene acetic acid): 0.93 mg/mL, IAA (indole-3-acetic acid): 1 mg/mL, 2,4-D (2,4-dichlorophenoxyacetic acid): 1 mg/mL , AS (Acetosyringone): 19.62 mg/mL.
